# Genome wide association and gene enrichment analysis reveal membrane anchoring and structural proteins associated with meat quality in beef

**DOI:** 10.1186/s12864-019-5518-3

**Published:** 2019-02-21

**Authors:** Joel D. Leal-Gutiérrez, Mauricio A. Elzo, D. Dwain Johnson, Heather Hamblen, Raluca G. Mateescu

**Affiliations:** Department of Animal Sciences, University of Florida, Gainesville, FL USA

**Keywords:** Protease substrate, Transmembrane proteins, Meat quality, And gene enrichment

## Abstract

**Background:**

Meat quality related phenotypes are difficult and expensive to measure and predict but are ideal candidates for genomic selection if genetic markers that account for a worthwhile proportion of the phenotypic variation can be identified. The objectives of this study were: 1) to perform genome wide association analyses for Warner-Bratzler Shear Force (WBSF), marbling, cooking loss, tenderness, juiciness, connective tissue and flavor; 2) to determine enriched pathways present in each genome wide association analysis; and 3) to identify potential candidate genes with multiple quantitative trait loci (QTL) associated with meat quality.

**Results:**

The WBSF, marbling and cooking loss traits were measured in *longissimus dorsi* muscle from 672 steers. Out of these, 495 animals were used to measure tenderness, juiciness, connective tissue and flavor by a sensory panel. All animals were genotyped for 221,077 markers and included in a genome wide association analysis. A total number of 68 genomic regions covering 52 genes were identified using the whole genome association approach; 48% of these genes encode transmembrane proteins or membrane associated molecules. Two enrichment analysis were performed: a tissue restricted gene enrichment applying a correlation analysis between raw associated single nucleotide polymorphisms (SNPs) by trait, and a functional classification analysis performed using the DAVID Bioinformatic Resources 6.8 server. The tissue restricted gene enrichment approach identified eleven pathways including “Endoplasmic reticulum membrane” that influenced multiple traits simultaneously. The DAVID functional classification analysis uncovered eleven clusters related to transmembrane or structural proteins. A gene network was constructed where the number of raw associated uncorrelated SNPs for each gene across all traits was used as a weight. A multiple SNP association analysis was performed for the top five most connected genes in the gene-trait network. The gene network identified the *EVC2*, *ANXA10* and *PKHD1* genes as potentially harboring multiple QTLs. Polymorphisms identified in structural proteins can modulate two different processes with direct effect on meat quality: in vivo myocyte cytoskeletal organization and postmortem proteolysis.

**Conclusion:**

The main result from the present analysis is the uncovering of several candidate genes associated with meat quality that have structural function in the skeletal muscle.

**Electronic supplementary material:**

The online version of this article (10.1186/s12864-019-5518-3) contains supplementary material, which is available to authorized users.

## Background

Beef consumers are highly interested in meat quality which is a complex of traits including tenderness, juiciness and flavor [1–3]. To respond to consumer demand, beef producers and retailers are interested in providing a consistent high-quality product [4] which requires a reliable and efficient method of measuring the quality of the final product. Beef quality grade is used to categorize carcasses and communicate the quality to consumers. Quality grade is determined predominantly by marbling; however, marbling only explains 5% of the variation in product palatability across carcasses [5]. Marbling and WBSF were identified from an extensive set of carcass and meat composition traits to be the best predictors of eating quality [6]. All the components defining the meat quality complex are quantitative traits, controlled by many genes and impacted by environmental factors. Most of these component traits are difficult and expensive to measure and not available to measure until late in life or after the animal has been harvested. Such traits are impractical to improve through traditional phenotypic selection but are ideal candidates for genomic selection if genetic markers that account for a worthwhile proportion of the phenotypic variation can be identified.

Genome wide association (GWA) studies have revealed well-supported associations, but these analyses generally explain only a small proportion of the phenotypic variance [7] because they can only identify genetic variants with medium to large effect. McClure et al. (2012) [8] analyzed five *Bos taurus* breeds and reported that only 1.02 and 1.85% of the observed phenotypic variation in WBSF could be explained by variation in calpastatin and μ-calpain genes, respectively, two major genes extensively analyzed in relationships with tenderness. Additional approaches, such as gene enrichment, have been designed to help identify genes with small to medium effect in an effort to understand the genetic basis of complex traits like beef palatability and meat quality [9]. The objectives of this study were: 1) to perform a GWA analysis for WBSF, marbling, cooking loss, tenderness, juiciness, connective tissue and flavor; 2) to determine enriched pathways in each GWA analysis; and 3) to identify candidate genes with multiple QTLs associated with meat quality.

## Results

### Phenotypes

Additive genetic differences and heterosis effect for carcass and meat palatability traits were estimated in this population previously [10]. The estimated heritabilities for marbling, tenderness and WBSF were 0.50 ± 0.05, 0.47 ± 0.06 and 0.17 ± 0.03, respectively, and the additive genetic correlation between WBSF and tenderness was − 0.97 ± 0.01 [11]. Table 1 shows the basic statistics for the meat quality phenotypes available for this study.

**Table 1:** Basic statistics for meat quality phenotypes

	N	Mean	SD
WBSF (kg)	672	4.30	1.15
Marbling	672	425.57	90.03
Cooking Loss (%)	672	23.25	5.74
Tenderness	495	5.28	0.84
Juiciness	495	5.01	0.76
Connective tissue	495	5.81	0.83
Flavor	495	5.56	0.46

Mean and SD for WBSF, marbling, cooking loss and sensory panel tenderness, juiciness, connective tissue and flavor in *longissimus dorsi* in a Brahman-Angus crossbreed population

### Genomic regions detected by the genome wide association analysis

**Fig. 1:**
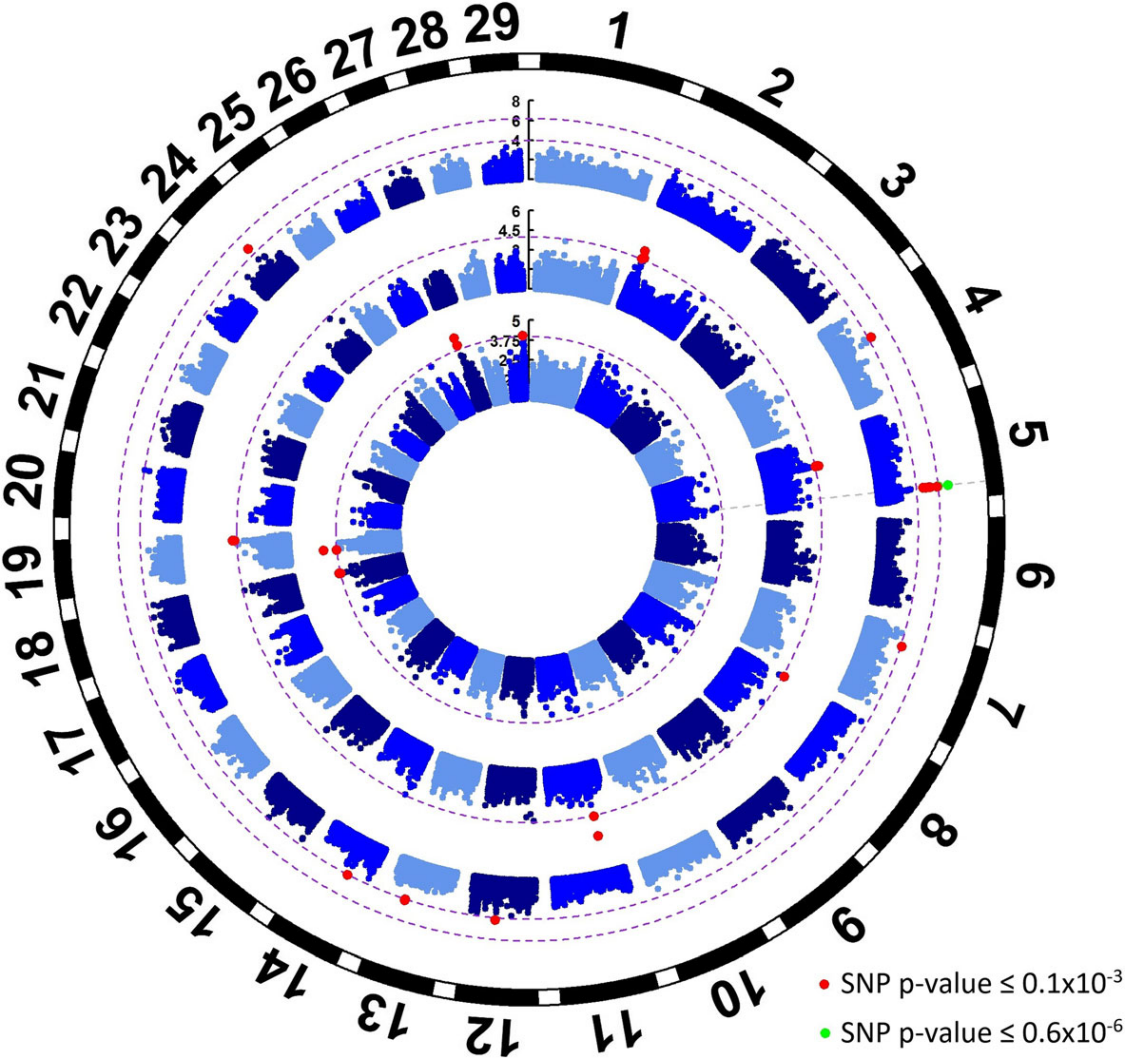
Genome wide association results for WBSF, marbling and cooking loss in *longissimus dorsi* muscle. From the outside to the inside, the rings represent a Manhattan plot for cooking loss, marbling and WBSF with -log10 *P*-values for 115,287 SNPs across the genome. Red dots represent SNPs with *p*-value lower than 0.1 × 10^− 3^ (first purple dotted line) and green dots represent SNPs with p-value lower than 0.6 × 10^− 6^ (second purple dotted line)

**Fig. 2:**
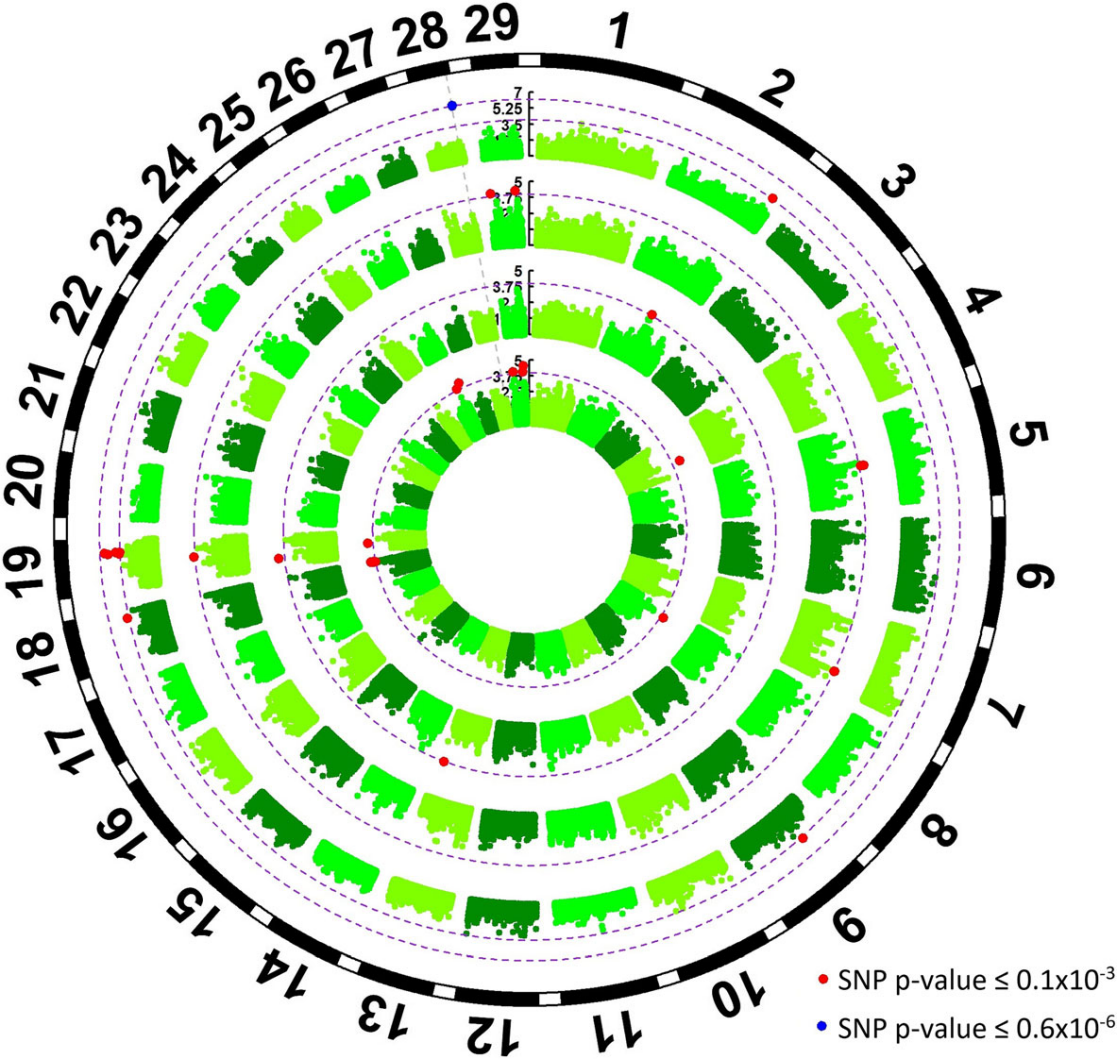
Genome wide association results for tenderness, juiciness, connective tissue, and flavor in *longissimus dorsi* muscle*.* From the outside to the inside, the rings represent a Manhattan plot for juiciness, tenderness, flavor and connective tissue with -log10 P-values for 115,287 SNPs across the genome. Red dots represent SNPs with p-value lower than 0.1 × 10^− 3^ (first purple dotted line) and blue dots represent SNPs with p-value lower than 0.6 × 10^− 6^ (second purple dotted line)

A total number of 68 genomic regions covering 52 genes were associated with meat quality traits (Additional file 1, and Figures 1 and 2) and 26.9% of these genes were involved in gene-expression, 15.4% with cell-signaling, 9.6% with cell-differentiation, 5.8% in apoptosis and 42.3% in other pathways.

### Enriched pathways related to meat quality

Ten pathways (Table 2) and eleven clusters (Additional file 2) were identified as enriched using the tissue restricted gene enrichment and the DAVID functional classification analysis approaches, respectively.

**Table 2:** Enriched gene ontology (GO) terms for meat quality traits measured in *longissimus dorsi* muscle

Trait	GO term	GO term name	p-value	Adjusted p-value	Number of genes in gene list	Number of genes in GO term
Marbling	GO:0003677	DNA binding	5.4 × 10^−3^	2.0 × 10^−1^	35	313
Connective tissue	GO:0005789	Endoplasmic reticulum membrane	1.0 × 10^−2^	3.6 × 10^− 1^	23	214
Cooking loss	GO:0005789	Endoplasmic reticulum membrane	1.4 × 10^−2^	4.8 × 10^−1^	22	214
Juiciness	GO:0005789	Endoplasmic reticulum membrane	4.3 × 10^−5^	**2.8 × 10** ^**−3**^	17	218
Tenderness	GO:0005789	Endoplasmic reticulum membrane	4.2 × 10^−3^	3.2 × 10^−1^	20	214
WBSF	GO:0005789	Endoplasmic reticulum membrane	2.1 × 10^−3^	1.9 × 10^−1^	22	213
Marbling	GO:0005794	Golgi apparatus	1.2 × 10^−2^	2.8 × 10^−1^	40	334
Cooking loss	GO:0005525	GTP binding	1.2 × 10^−2^	8.0 × 10^−1^	17	179
Marbling	GO:0005525	GTP binding	4.0 × 10^−2^	5.9 × 10^−1^	20	175
Connective tissue	GO:0005743	Mitochondrial inner membrane	1.3 × 10^−2^	3.0 × 10^−1^	17	168
Connective tissue	GO:0000122	Negative regulation of transcription from RNA polymerase II promoter	4.3 × 10^−4^	**3.0 × 10** ^**−2**^	19	225
Marbling	GO:0000122	Negative regulation of transcription from RNA polymerase II promoter	4.2 × 10^−3^	3.1 × 10^−1^	22	223
Connective tissue	GO:0045944	Positive regulation of transcription from RNA polymerase II promoter	1.9 × 10^−2^	3.4 × 10^−1^	40	319
Flavor	GO:0045944	Positive regulation of transcription from RNA polymerase II promoter	7.9 × 10^−4^	**6.2 × 10** ^**−2**^	35	325
Flavor	GO:0006355	Regulation of transcription, DNA-templated	7.1 × 10^−3^	2.8 × 10^−1^	36	299
Cooking loss	GO:0003723	RNA binding	4.2 × 10^−2^	9.6 × 10^−1^	15	145
Marbling	GO:0006351	Transcription, DNA-templated	1.3 × 10^−2^	2.4 × 10^−1^	38	318

Significance of each enriched term was adjusted using the Benjamini-Hochberg p-value correction

### Tissue restricted gene enrichment

**Fig. 3:**
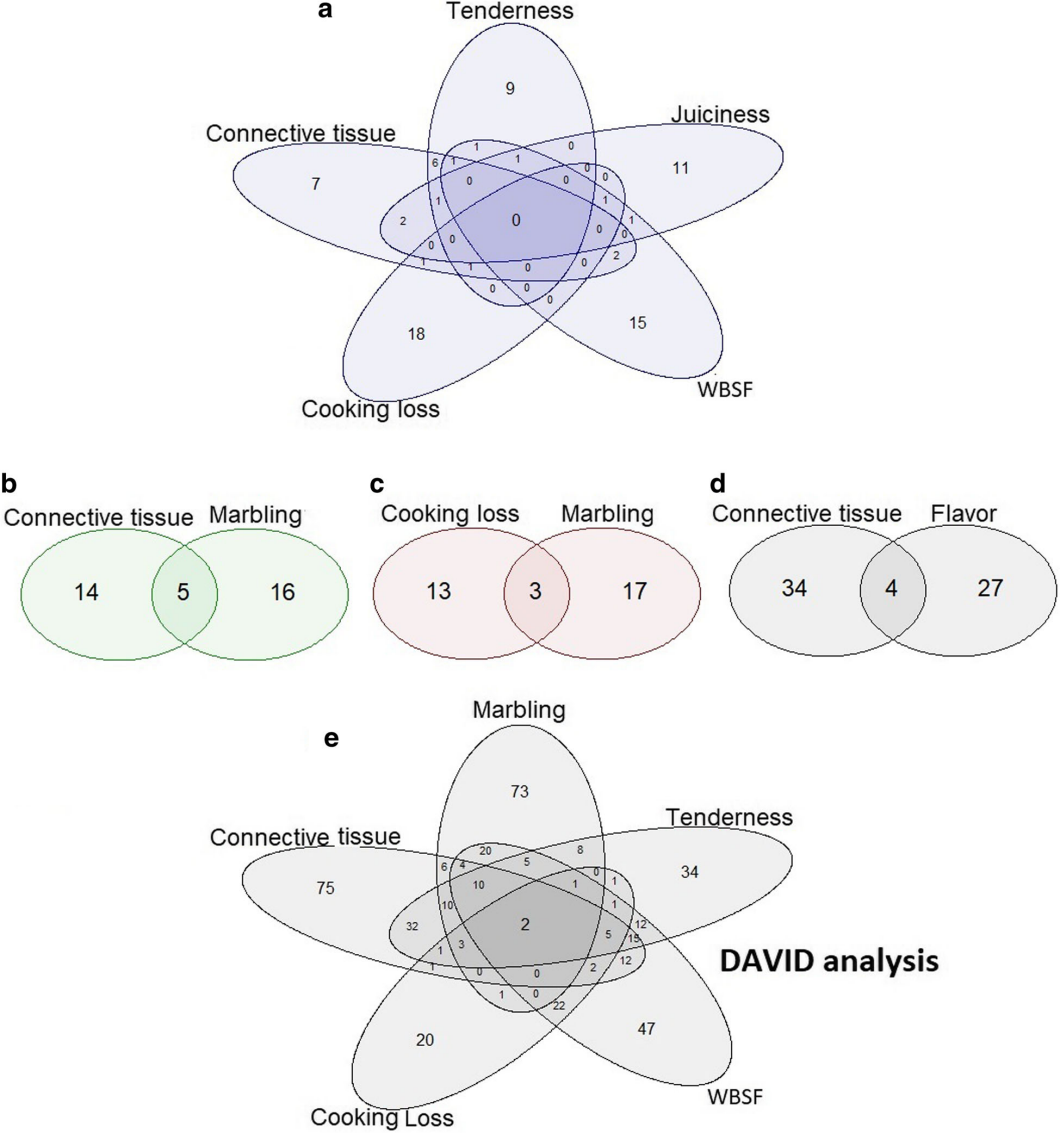
Number of genes enriched for meat quality traits in overlapping pathways. Pathways identified as enriched in more than one trait simultaneously following GWAs for meat quality traits using the tissue restricted gene enrichment approach (**a, b, c, d**) or a DAVID functional classification analysis (**e**)

The tissue restricted gene enrichment approach identified the “Endoplasmic reticulum membrane”, “Negative regulation of transcription from RNA polymerase II promoter”, “Positive regulation of transcription from RNA polymerase II promoter”, and “Guanosine-5’-triphosphate (GTP) binding” pathways for more than one trait simultaneously (Fig. 3). Additionally, six other pathways were identified as enriched for a single trait (Additional file 3).

### DAVID functional classification analysis

Twenty-one genes were identified through the functional classification analysis based on DAVID in at least four traits (Fig. 3e and Additional file 4). Many of these genes, such as *EPHA7*, *NRXN2*, *CADM1*, *PALLD*, and *VCAN*, encode integral components of membranes, or are protein extracellular matrix constituents.

### Gene network and candidate genes with multiple QTLs across meat quality traits

**Fig. 4:**
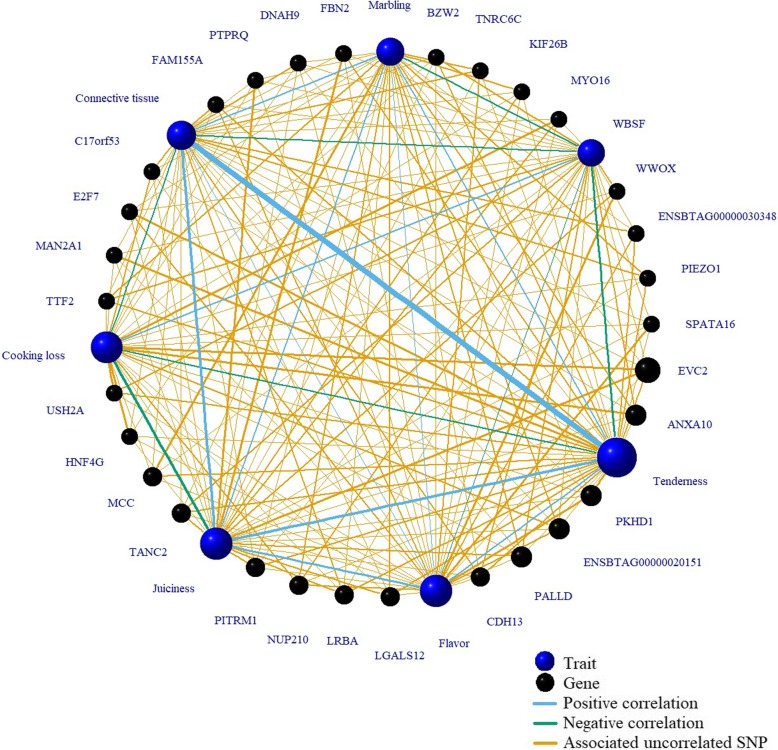
**N**etwork of genes with multiple associated uncorrelated SNPs with meat quality traits. Top 30 genes with the highest number of raw associated uncorrelated SNPs across Warner-Bratzler Shear Force (WBSF), marbling, cooking loss and sensory panel tenderness, juiciness, connective tissue and flavor. The size of the trait and gene nodes indicates the degree of connectivity. Positive (blue lines) and negative (green lines) represent the phenotypic correlation between traits with the thickness of the line indicating the strength of the correlation (ranging from − 0.43 to 0.83)

Figure 4 shows the top 30 genes with the highest number of raw associated uncorrelated SNPs across all traits. The top five genes in the network had a significantly higher connectivity than the rest of the genes and they are discussed below.

## Discussion

### Phenotypes

The average marbling score was 425.57 ± 90.03 which is comparable to the national beef industry average quality [12] and similar to previously reported data in this multibreed population [10, 13, 14]. Based on the taste panel measurements, steaks were classified on average as slightly tender and slightly juicy. Flavor was moderately intense and only traces of connective tissue were perceived by panelists during the sensory panel.

### Genomic regions detected by the genome wide association analysis

#### Genomic regions associated with WBSF

Four chromosomal regions were associated with WBSF and harbored the *RWDD4A*, *NUMBL*, *BLMH*, *SMG6* and *LRP5* genes. The LRP5 is a cell-surface co-receptor of Wnt/beta-catenin signaling and has a pivotal role in bone formation. Polymorphisms in LRP5 can modulate the relationship between physical activity and bone density suggesting that LRP5 may play a role in bone mechanical load adaptation. Thus, LRP5 could be involved in bone/muscle cross-talk through the Wnt signaling [15]. Basic and clinical research on bone metabolism and muscle biology suggests that bone interacts with skeletal muscle via signaling from local and humoral factors [16]. A chromosomic region harboring *LRP5* was associated with fatty acid composition and WBSF in *longissimus* muscle from *Bos taurus* [17, 18].

A number of studies have reported associations of polymorphisms in μ-calpain and calpastatin with tenderness, and several of these polymorphisms had a large effect in *Bos taurus* populations [8, 18–20]. In the present study, no association was found between meat quality related traits and μ-calpain or calpastatin, which could be explained by the *Bos indicus* influence [21]. This reconfirms that genetic markers discovered in *Bos taurus* populations are usually not predictive in *Bos indicus* populations most likely due to changes in size effect and stringent *p*-value corrections applied in GWA approaches. In the same population used in this study, Leal-Gutiérrez et al. [22] reported multiple polymorphisms in the 3′ region of calpastatin associated with WBSF, however, none of them had a large individual effect. Additionally, in the same population, Wright et al. [23] found that μ-calpain autolysis was related to troponin-T, desmin, and titin degradation, and these were related to variation in WBSF and taste panel tenderness.

#### Genomic regions associated with marbling

Three genomic regions (2:7,343,971 – 7,437,564, 5:47,654,327 – 47,827,349, 11:27,755,975 – 27,894,797, and 19:56,569,168 – 56,584,509) had at least two SNPs associated with marbling. The *COL3A1* gene located on BTA2 encodes a collagen protein that is more abundant in soft connective tissues [24]. Higher fat and collagen deposition in skeletal muscle is observed in Duchenne muscular dystrophy patients, and it has been suggested that a common mesenchymal progenitor cell is responsible for fat and fibrosis accumulation in skeletal muscle. This relationship between fat and fibrosis accumulation could impact meat quality perception. However, it is unclear how adipocytes or collagen-producing cells are regulated before differentiation from mesenchymal progenitors [25]. Other previously reported genes of this family associated with marbling are: *COL11A1*, *COL15A1*, *COL1A1*, *COL24A1*, *COL28A*, *COL2A1*, *COL4A3*, *COL6A3* and *COL23A1* [19, 21].

Four genes (*GRIP1*, *HELB*, *FAU* and *IRAK3*) are located on BTA5 between 47,654,327 – 47,827,349. With the exception of *FAU,* all other genes had at least one SNP associated with marbling. The GRIP1 is a steroid receptor coactivator involved in nuclear receptor-mediated activation of transcription. It activates myogenin, MEF-2, p21, and also promotes expression of contractile proteins and myotube formation. Thus, GRIP1 is involved in myocyte differentiation [26–28]. The *HELB* encodes a 5–3 DNA Helicase important for DNA damage response [29]. The *SRBD1* and *ENSBTAG00000032651* genes are located inside the 11:27,755,975 – 27,894,797 chromosomic region which has two associated SNPs. No conclusive biological support for this association was evident; nevertheless, neighboring regions have been previously reported as associated with fatty acid composition in *longissimus* muscle from *Bos taurus* and *Bos indicus* cattle [17, 30].

One region on BTA19 harboring *RECQL5*, *SMIM5* and *ENSBTAG00000011713* genes was associated with marbling. The RECQL5 is involved in DNA replication, transcription and repair. The role of RECQL5 in transcription has been related to inhibition of stalled transcript elongation at damaged DNA sites by binding to the RNA polymerase II [31]. The *SMIM5* and *ENSBTAG00000011713* encode structural proteins in skeletal muscle. This region in the BTA19 has been previously reported as associated with carcass and meat quality, fatty acid composition, and mineral and peptide content [17, 18].

#### Genomic regions associated with cooking loss

A genomic region on BTA5 (105,049,390 - 105,291,830) showed a highly significant association with cooking loss. Two SNPs in this region were significant at the genome wide *p*-value threshold, while other five SNPs were significant at the 0.1 × 10^− 3^ threshold. Two genes are located in this region: *ANO2* and *NTF3* (Fig. 1). The ANO2 is an intracellular calcium activated chloride channel [32]. The NTF3 belongs to the Neurotrophic Factor family which modulates survival and function by innervating motoneurons and proprioceptive neurons. The Neurotrophic Factor family is also involved in myoblast and muscle fiber development and differentiation, muscle innervation coordination and functional differentiation of neuromuscular junctions [33]. Mice lacking one *NTF3* functional gene copy had smaller cross-sectional fiber area, and fibers were more densely packed than in the wild type phenotype. Adult *NTF3* deficient mice showed weaker movement compared to wild type when exposed to electrical stimulation [34]. These results indicate that NTF3 is involved in nerve terminal maturation and synaptic vesicle recycling, and changes to these processes can lead to changes in muscle fiber diameter. The Additional file 5 shows the *ANO2-NTF3* region in detail and the SNPs associated with cooking loss. Although the associated SNPs are evenly distributed outside and inside several LD-blocks, they are highly correlated, as shown on the SNP correlation heat map. The distribution of these SNPs suggests the presence of a single, unique QTL for cooking loss located downstream from *ANO2*. When the most significant SNP in this region, rs137723969, was fitted as a fixed effect in the GWA analysis, all remaining SNPs inside this region lost their significance. The rs137723969 SNP is a T/C intronic substitution, thus, it could have a regulatory function or it might be in strong LD with the functional polymorphism.

Two other genomic regions on BTA 4 and BTA 13 were associated with cooking loss. The BTA 4 region 40,441,870 - 40,454,953 had two associated SNPs located in the *ENSBTAG00000047646* gene. This gene is the bovine orthologue of the human *CD36*. The CD36 is a multifunctional glycoprotein, and its molecular function is to be a receptor for a broad range of ligands, from fibronectin and collagen to low-density lipoprotein, anionic phospholipids, and long-chain fatty acids [35]. The CD36 regulates plasma membrane fatty acid transport and has been found responsible for an increased passage of fatty acids in obese and insulin resistant people. This higher rate of transport contributes to the increased accumulation rates of triacylglycerol in skeletal muscle through a critical role in regulation of fatty acid esterification and oxidation performed by CD36 [36]. The association of this region with cooking loss could be related to changes in fatty acid deposition and/or composition and its loss as fluid during cooking. Mateescu, Garrick, & Reecy [18] reported association of a region located 0.6 megabases downstream of *CD36* with a panel of carcass and meat quality traits, mineral and peptide content and fatty acid composition. The second region is located between 13:60,209,040 and 13:60,210,256, and three associated SNPs are located inside the *SIRPD*; however, no conclusive biological support was evident.

The *CHSY3* and *PREX2* genes were also found associated with cooking loss. The former gene encodes a transmembrane protein associated to the Golgi apparatus. Dang et al. [37] and Chen et al. [17] found association of chromosomic regions close to *CHSY3* and *PREX2* with tenderness and fatty acid content in *Bos taurus*, respectively.

#### Genomic regions associated with tenderness

Four genes (*CFAP54*, *GPR98*, *TUT1* and *SLC47A1*) were associated with sensory tenderness. The CFAP54 and GPR98 are integral components of structural proteins and membranes. The *GPR98* has been associated with bone density, levels of serum osteocalcin, bone formation marker, and urine deoxypyridinoline in human and confirmed in mice [38]. Some *GPR98* neighboring regions have been reported as associated with multiple carcass and meat quality traits, and fatty acids composition. The biological function of TUT1 is related to synthesis of the 3-poly(A) tail and 3’end cleavage of specific pre-mRNAs, and this region has been reported as associated with fatty acid composition [17, 39].

#### Genomic regions associated with juiciness

One marker located at 28:41,901,391 and mapped to the *MMRN2* gene was highly associated with juiciness. This gene encodes an extracellular matrix glycoprotein, and although its biological role has remained elusive, it is believed to be a key component in endothelial cell function regulation, neoangiogenesis and tumor growth [40]. Chen et al. [17] and Castro et al. [41] reported association of neighboring regions to *MMRN2* with fatty acid composition and WBSF in *longissimus* muscle from *Bos taurus* and *Bos indicus*.

Four SNPs in regions 45,889,900 – 45,964,845 and 47,514,682 – 47,523,394 on BTA19 were associated with juiciness. The *ENSBTAG00000044940* and *GOSR2* genes are located inside the first region while *METTL2* gene is located inside the second. The *GOSR2* gene is involved in the transport of critical membrane glycoprotein complexes in myocytes [42]. In humans, double Gly144Trp mutants in GOSR2 had progressive cortical myoclonus and ataxia with areflexia, showing reduced motor unit recruitment given chronic partial denervation [43]. A *GOSR2* neighboring region has been reported as associated with oleic acid content in Japanese Black cattle [44]. One exonic SNP in *LIG1* was found associated with juiciness, and a window including this region has been previously reported as associated with WBSF [8].

#### Genomic regions associated with connective tissue

One region on BTA18 was associated with the connective tissue amount, and this region harbors *CNOT3*, *OSCAR* and *NLRP5* genes. The *CNOT3* is a component of the CCR4-NOT complex. This complex codes for major mRNA deadenylases, and it is linked to numerous cellular processes such as miRNA-mediated repression, bulk mRNA degradation, general transcription regulation and translational repression during translational initiation [45]. Morita et al. [46] documented that expression of two CNOT3 regulated genes, *PDK4* and *IGFBP1*, are increased in Cnot3+/− hepatocytes since they have longer poly(A) tails than those seen in the control. Additionally, the increased expression of CNOT3 target genes was associated with greatly decreased visceral and subcutaneous fat deposition in Cnot3+/− mice. The *OSCAR* is an osteoclastogenesis regulator and plays an important bone-specific function in osteoclast differentiation [47]. The *ATRNL1* and *NFIB* were found associated with connective tissue, and Chen et al. [17] and Saatchi et al. [48] reported association of a chromosomic region close to these genes with fatty acid composition in *Bos taurus* cattle.

#### Genomic regions associated with flavor

Flavor had three associated SNPs in *MARCO*, *ZMYND8* and *ACCN1*. The ZMYND8 may act as a transcriptional corepressor of the *KDM5D*, and since KDM5D is a histone demethylase that specifically demethylates Lys-4 of histone H3, ZMYND8 plays a central role in histone modifications [49]. Chen et al. [17] reported association of a 0.4 megabases downstream region of the *ACCN1* associated SNP with fatty acid composition in *Bos taurus*; nevertheless, no biological support for this association was found.

### Enriched pathways related to meat quality

#### Tissue restricted gene enrichment

The endoplasmic reticulum (ER) membrane pathway was enriched for WBSF, cooking loss, tenderness, juiciness, and connective tissue. Figure 3a shows the number of genes in each enriched pathway for each trait. Five genes (*SEC16B*, *REEP1*, *SLC8A3*, *TMEM147* and *CD4*) were identified in the ER membrane pathway for at least three traits simultaneously. The *SEC16B* is required for secretory cargo traffic from the ER to the Golgi apparatus and for normal transitional ER organization [50]. The REEP1 gene encodes a transmembrane protein required by ER-cytoskeleton network formation, shaping and remodeling [51]. The electrogenic exchange of Ca(2+) against Na(+) ions across the cell membrane is performed by SLC8A3 and contributes to the cytoplasmic regulation of Ca(2+) levels, Ca(2+)-dependent cellular processes and to cellular Ca(2+) homeostasis in excitable cells such as myocytes. The SLC8A3 gene was included in the “transmembrane signaling receptor activity” and “extracellular matrix structural constituent” GO terms [52, 53].

Many proteins in this pathway are structural constituents of the ER, and their implication in meat quality could be hypothesized through changes in ER macrostructure and cytoskeletal binding. Some human hereditary spastic paraplegias result from mutations in *REEP1* which is required for ER network formation [51]. The REEP1 is structurally related to the DP1/Yop1p family of ER-shaping proteins, and it colocalizes and forms protein complexes with spastin and atlastin-1. Hydrophobic residues in REEP are critical in supporting membrane curvature [54]. A mutant REEP1 protein lacking the C-terminal cytoplasmic region, disrupts the ER network in vitro*,* demonstrating that REEP1 binds to microtubules and aligns along the microtubule cytoskeleton [51]. Overexpression of *REEP1* dramatically alters ER morphology because this protein has ER-shaping and ER-remodeling function by interacting directly with microtubules [51]. The “Endomembrane system” and “Golgi apparatus” GO terms was identified as enriched following a GWA analysis for WBSF in Nelore cattle [41], showing the importance of structural proteins for the cytoskeletal myocyte framework and tenderness. Proteins in this pathway could be related to proteolysis. The tenderization process is determined by the amount of disruption of cytoskeletal proteins such as desmin, metavinculin, nebulin, dystrophin and vinculin by μ-calpain [55–58]. Nevertheless, transmembrane proteins such as TMEM147 and CD4 could be membrane anchors of the cytoskeletal proteins to the plasma and organelle membranes, thus their proteolysis may be critical for tenderization. Giving that REEP1 is not a μ-calpain substrate (Additional file 6), this protein may be important for cellular compartmentalization or increased cytoskeletal stability after aging.

The *ZMYND11*, *PDE2A*, *GLIS2*, *GLI2*, and *HMGA2* genes from the “negative regulation of transcription from RNA polymerase II promoter” pathway were enriched for connective tissue and marbling. Three genes (*CDH13*, *NKX3–1*, and *PARK2*) from the “positive regulation of transcription from RNA polymerase II promoter” pathway were also identified as enriched for connective tissue and flavor simultaneously (Fig. 3b and d). The ZMYND11 is a chromatin reader, and it specifically recognizes and binds to histone 3. It regulates RNA polymerase II elongation and colocalizes with highly expressed genes acting as a transcription corepressor by restraining RNA polymerase II at the elongation stage [59]. The *PDE2A* has phosphodiesterase activity, and it is involved in cAMP (cyclic adenosine 3′:5′-monophosphate) catabolism and signal transduction [60]. The GLIS2 and GLI2 are hedgehog signaling pathway regulators, and HMGA2 modulates transcriptional processes and plays a role in postnatal myogenesis and satellite cell activation [61]. The *CDH13* encodes a protein localized to the surface of the cell membrane, and it is hypermethylated in some types of cancer, the NKX3–1 transcription factor behaves as a repressor, and PARK2 protein can repress p53/TP53 protecting against apoptosis [62].

The relation of these two pathways with meat quality traits can be explained through changes in the expression of genes related to connective and adipose tissue deposition. A nephronophthisis-like phenotype in human and mice was found associated with a mutation in the *GLIS2* gene [63], with severe renal fibrosis and atrophy resulting from upregulation of fibrosis related genes and increased apoptosis in the *GLIS2* mutant kidneys. Overexpression of *HMGA2* has been found in activated myosatellite cells; however, gene expression declines significantly as fusion of myoblasts into myotubes proceeds and also during muscle regeneration [64, 65]. It has been documented that *HMGA2* is located inside a QTL for lean mass percentage, growth and back fat in *longissimus* muscle from swine [66].

The *ERAL1*, *GMPPB* and *LRRK2* genes were enriched in the GTP binding pathway for cooking loss and marbling (Fig. 3c). The *ERAL1* encodes a GTPase involved protein required for assembly of the mitochondrial ribosomal small subunit. The LRRK2 has GTPase activity [67]. Some dominant mutations in the human *GMPPB* associated with defects in protein glycosylation have been identified [68]. These defects result in morphological changes in the neuronal membrane, and dystrophic changes are evident with ring fibers, increased fibrosis, necrosis, and multiple regenerating fibers and abnormal neuromuscular transmission in muscle. Some of these mutations lead to abnormal GMPPB folding, which results in cytoplasm protein aggregates [69]. An associated cluster of GDP (Guanosine-5′-diphosphate)-GTP (Guanosine-5′-triphosphate) conversion related genes on BTA5 and “GTPase binding” enrichment have been reported for meat quality in Nelore populations [41, 70].

#### DAVID functional classification analysis

The *EPHA7* and *NRXN2* genes were found across all five phenotypes and are expressed in bovine skeletal muscle. The *EPHA7* encodes a tyrosine kinase receptor involved in cell signaling and modulation of cell-cell adhesion [71]. Vickerman et al. and Lai et al. [72, 73] showed that EPHA7 is co-localized at the neuromuscular junction of adult muscle being physically associated with the actin cytoskeleton. Changes in *NRXN2* expression in motor neurons have been reported as the cause of spinal muscular atrophy in mammals [74]. Only four genes in the ER membrane pathway were found in common between the tissue restricted gene enrichment analysis and the DAVID functional classification analysis (*TYRO3*, *BCL2*, *POMGNT2* and *PTGS1*). The TYRO3 is an integral component of plasma membrane, ER membrane and nucleus. The BCL2 is not a membrane bound protein but it is found in mitochondrion and ER. Both TYRO3 and BCL2 are involved in cell survival and apoptosis. The POMGNT2 protein is an integral component of the ER membrane with transferase activity.

### Gene network and candidate genes with multiple QTLs across meat quality traits

The *EVC2* gene had the highest connectivity in the network. Additional file 7 shows the SNP association plot and the LD-block prediction for this gene. The *EVC2* encodes a positive regulator of the hedgehog signaling pathway, and it has a critical role in skeletal development and bone formation [75]. Hedgehog signaling modulates patterning and morphogenesis of most organs in mammalian embryo, and mutations in *EVC* or *EVC2* disrupt the hedgehog signaling in bone development, with a high proportion of Ellis-van Creveld syndrome patients presenting mutations in *EVC2* [76]. Tenderness, cooking loss and juiciness had two, three and three raw associated uncorrelated SNPs in *EVC2*, respectively. When fitted together in the association model, only one SNP was significant for tenderness, two for cooking loss (rs455584405 and rs43488309) and other two SNPs (rs43488309 and rs383679972) for juiciness (Additional files 7 and 8). The LD-block prediction for *EVC2* revealed that these pairs of SNPs significant for each trait were located in different LD-blocks. This suggests that different segments of *EVC2* contribute independently to the phenotypic variability present in cooking loss and juiciness in the present population.

The bioinformatic analysis of these SNPs revealed that the C allele of rs455584405 reduces the Minimum Free Energy (MFE) parameter by 4.1 kcal/mol, which was the largest impact on MFE from the entire set of SNPs. The missense rs455584405 SNP produces a E6D change in the EVC2 protein. The EVC2 is a transmembrane protein, with a cytoplasmic portion encoded by the 1 to 196 amino acids, the transmembrane portion located between the amino acids 197 and 221, and the remaining representing the extracellular region. No signal peptide was predicted for this protein and the E6D change does not have an effect on the EVC2 isoelectric point. Thus, a possible change in the interaction between the cytoplasmic portion of EVC2 and other cytoskeletal compounds could explain this association. The rs43488309 marker is a synonymous SNP, and the C > T change produces a reduction of 3.2 kcal/mol in MFE and minimal changes in mRNA folding.

The *ANXA10* and *PALLD* are contiguous genes located on BTA8 (Additional file 9). *ANXA10*, a constituent of the multigene Annexins family, is a Ca2 + −regulated phospholipid- and membrane-binding protein. This family has been described as involved in diverse biological mechanisms from the control of membrane structure to certain membrane transport processes [77]. The *PALLD* gene encodes a cytoskeletal protein essential for normal cytoskeletal organization and involved in establishing cell morphology, cell adhesion and motility, and cell-extracellular matrix interactions. PALLD functions as a scaffolding molecule affecting actin polymerization and actin filament assembly [78]. *ANXA10* showed a higher LD level that the *PALLD* locus. This might suggest that more markers are required to map QTLs in *PALLD*. A total of seven SNPs in *ANXA10* were identified as raw associated uncorrelated SNPs for WBSF (2 SNPs), marbling (2 SNPs) and cooking loss (3 SNPs). However, only two SNPs for WBSF, and only one SNP for marbling and cooking loss are required to explain all the variability in the *ANXA10-PALLD* region. The two indels (rs110953884 SNP and rs464042833) significant for WBSF are located in intronic regions of *ANXA10*; thus, they could map neighboring functional polymorphisms or have a regulatory function in this gene.

The *PKHD1* gene (Additional file 10) encodes a membrane and cytoskeleton associated binding protein related to the “positive regulation of cell proliferation” and “single organismal cell-cell adhesion” pathways. Several phenotypes are associated with mutations in *PKHD1*: congenital hepatic fibrosis, biliary tract abnormality and absence of renal corticomedullary differentiation [79–81]. Although this gene has not been associated with any skeletal muscle related phenotype, it is expressed at a low level in skeletal muscle [82]. The *PKHD1* locus is in a low LD region with the possibility of multiple QTLs. All three raw uncorrelated SNPs associated with WBSF were significant when fitted simultaneously, as was the case with the two significant SNPs for flavor. The existence of several associated SNPs in different LD-blocks of *PKHD1* indicate that different regions of this gene could contribute independently to tenderness and flavor in the present population.

The PKHD1 protein contains an intracellular segment (3873–4071 amino acids), a transmembrane segment between 3850 and 3872, and an extracellular segment. The bioinformatics analysis predicted a signal peptide between amino acids 1–17. Three SNPs (rs43559996, rs209661307 and rs714029807) were significant when fitted simultaneously for tenderness, while two other SNPs (rs208145052 and rs718439487) were significant for flavor. The bioinformatic analysis identified only 2 of these SNPs (rs43559996 and rs209661307) as able to modify the MFE parameter (by 3.4 and 1.3 kcal/mol, respectively) and have the biggest impact on mRNA folding. All five SNPs are missense polymorphisms (Additional file 8), and they are able to change the isoelectric point parameter by 0.01. The rs714029807, rs718439487, rs208145052, rs209661307 SNPs are located in the extracellular segment of PKHD1 and rs43559996 is found in the cytoplasmic portion. Thus, all these SNPs might affect the physical relationship between PKHD1 and the extracellular matrix and other cytoskeletal proteins.

*ENSBTAG00000020151* gene codes for a protein with nucleic acid binding activity [83] (Additional file 11), but its specific function has not been clearly identified. Two associated uncorrelated SNPs associated with marbling are located inside the first LD block; however, only one is able to explain all the variability present in this region when both are fitted simultaneously in the model. Two uncorrelated SNPs in this gene were found associated with cooking loss and flavor but in both cases one SNP is able to explain all the variability present in each trait.

### Biological mechanisms associated with meat quality

Multiple myocyte structural proteins and related pathways were identified in the present analysis. From the 52 genes identified in the GWA approach, 48% encode transmembrane proteins or membrane associated molecules and 5.7% encode cytoskeletal proteins. Genes in the Endoplasmic reticulum membrane, Golgi apparatus, GTP binding and Mitochondrial inner membrane were enriched, and clustering of the same type of proteins was evident using the functional classification analysis. Polymorphisms in genes coding for structural proteins can modulate two different processes with direct effect on meat quality: in vivo myocyte cytoskeletal organization and postmortem proteolysis.

From the genes associated with the in vivo myocyte cytoskeletal organization, EHD, BNIP3, MARCO and COL5A1 are the most interesting and are discussed further. The *EHD* gene is involved in receptor and lipid recycling in plasma membrane and promotes cytoskeletal reorganization and tubule formation; EHD also physically interacts with myoferlin, a protein involved in myoblast fusion [84]. The myoblasts of the EHD1 knockout mice showed impaired receptor recycling and key muscle protein misplacement [84], resulting in muscles with reduced myoblast fusion, smaller myofibers and overgrown T-tubules. Defective EHD1 adipocytes have enlarged endosomes and cytoplasmic dispersion of perinuclear GLUT4-containing membranes [85]. It was reported that EHD1 regulates transport of β1-integrin, a protein involved in multiple signaling pathways and a key for processes such as proliferation, apoptosis, cell spreading and migration [86]. EHD1 knockdown produces impaired β1-integrin recycling and accumulation in a transferrin-containing endocytic recycling compartment. Fibroblastic plasma membranes from EHD1 knockouts had lower content of β1-integrin, more prominent focal adhesions, and diminished migration and cell spreading capacity. Activation and upregulation of the *MARCO* gene is associated with changes in actin cytoskeleton organization during cellular splenic maturation. Immature splenic dendritic cells are adherent and show visible actin structures while mature cells are nonadherent, round and have punctate actin cytoskeletal structure [87]. The *BNIP3* modulates actin cytoskeleton plasticity and knockdown of this gene results in a more stable tubular-like network, different actin cytoskeletal remodeling activity, upregulated formation of actin stress fibers, decreased lamellopodial protrusions and filopodia [88]. In wild type fibroblasts, COLLV and COLLIII are arranged as large fibrils in the intercellular spaces but mutations in COL5A1 and COL3A1 alter collagen organization in the extracellular matrix and affect clustering of α2β1 integrin receptor. Mutations in COL5A1 and COL3A1 also promote collagen retention in the cytoplasm [89].

Polymorphisms in other structural proteins are associated with functional alterations. Yadav et al. [90] reported that four mutations in *GLURΔ1* trigger spontaneous gate opening. Mutations in *C19ORF12* gene modifies protein location and function; *C19ORF12* encodes a protein located in the mitochondrial and endoplasmic reticulum membrane and mutations in this gene impair protein subcellular location and cellular response to oxidative stress [91]. Activating mutations in *PDGFRA* have been identified in patients with gastrointestinal stromal tumors. Mutations in the PDGFRA transmembrane domain simulates dimer transmembrane domain packing and promotes cell growth and signaling in the absence of ligand [92].

The second mechanism is associated with the amount of structural protein disruption in the myocyte after aging. Meat quality is mainly determined by the extent of postmortem proteolysis of key cytoskeletal, cytoskeletal-associated proteins, and extracellular matrix-associated proteins. This proteolysis is accomplished by degradation of structural proteins such as desmin and talin during aging through the activity of the endogenous μ-calpain-calpastatin system [93–96]. Filaments such as desmin and talin are the main substrates of this endogenous system, but membrane anchoring proteins identified in the present analysis might have a significant contribution. From a total of 72 structural proteins identified as associated with meat quality traits in the present analysis (Additional file 6), 87.5% of these proteins are potential substrates of μ-calpain. Some previously reported membrane associated proteins and calpain substrates are: GIUR1, NR2A, CFTR and ITPR1 [97–101]. Many of these proteins anchor cytoskeletal filaments to the sarcolemma and organelle membranes, therefore the lysis of these proteins during proteolysis could have a direct effect on overall cytoskeletal stability.

## Conclusion

A total number of 68 genomic regions covering 52 genes were associated with meat quality traits and these genes are related to gene-expression, cell-signaling, cell-differentiation, and apoptosis; 48% of these genes encode transmembrane proteins or membrane associated molecules and 5.7% encode cytoskeletal proteins. A tissue restricted gene enrichment identified two main kinds of pathways: pathways associated with membrane structural proteins such as Endoplasmic reticulum membrane, Golgi apparatus, GTP binding and Mitochondrial inner membrane, and pathways related to gene expression including Negative regulation of transcription from RNA polymerase II promoter, Positive regulation of transcription from RNA polymerase II promoter, Regulation of transcription, DNA-templated, RNA binding and Transcription DNA-templated. A DAVID functional classification analysis identified clusters mainly related to integral components of membranes. A gene network identified the *EVC2, ANXA10* and *PKHD1* as potential genes with multiple QTLs associated with meat quality.

Polymorphisms in structural proteins can modulate two processes with direct effect on meat quality: in vivo myocyte cytoskeletal organization and postmortem proteolysis. Gene related to the first process such as EHD, BNIP3, MARCO and COL5A1 control cytoskeletal organization and remodeling, T-tubules growth and transport of other transmembrane proteins. During postmortem proteolysis, structural proteins are disrupted in the myocytes. Filaments such as desmin and talin are the main proteolysis substrates, but membrane anchoring proteins identified as associated in the present analysis might have a significant contribution. Out of 72 structural proteins identified as associated with meat quality traits in the present analysis, 87.5% are potential substrates of μ-calpain. Lysis of these proteins during proteolysis could have a direct effect on overall cytoskeletal stability giving that many of these proteins anchor cytoskeletal filaments to the sarcolemma and organelle membranes.

## Methods

### Cattle population and phenotypic data

The research protocol was approved by the University of Florida Institutional Animal Care and Use Committee number 201003744. The animals were born between 2007 and 2014 and belong to the multibreed Angus-Brahman herd from the University of Florida [102]. Cattle were classified into six different groups based on their expected Angus and Brahman breed composition. Based on the Angus composition, the grouping was as follows: 1 = 100 to 80%; 2 = 79 to 65%; 3 = 62.5% (Brangus); 4 = 59 to 40%; 5 = 39 to 20%; 6 = 19 to 0%.

When steers reached 1.27 cm subcutaneous fat thickness over the ribeye, they were transported to a commercial packing plant with an average slaughter weight of 537.94 ± 55.31 kg at 17.31 ± 1.23 months. A total number of 672 steers were harvested using established USDA-FSIS procedures, and after 48 h postmortem, marbling was recorded by graders’ visual appraisal of the ribeye muscle at the cut surface after the carcass had been ribbed at the 12th/13th rib interface. The marbling grade was as follows: Practically Devoid = 100–199, Traces = 200–299, Slight = 300–399, Small = 400–499, Modest = 500–599, Moderate = 600–699, Slightly Abundant = 700–799, Moderately Abundant = 800–899, Abundant = 900–999. A Johnson normalization was applied to marbling [103].

Two 2.54 cm steaks from the *longissimus dorsi* muscle at the 12th/13th rib interface were sampled from each animal. Steaks were transported to the Meat Science Laboratory of the University of Florida, aged for 14 days at 1 to 4 °C, and then stored at − 20 °C. Both frozen steaks from each animal were allowed to thaw at 4 °C for 24 h and cooked to an internal temperature of 71 °C on an open-hearth grill. After cooking, one steak was cooled at 4 °C for 18 to 24 h and used to measure WBSF and cooking loss according to the American Meat Science Association Sensory Guidelines [104]. Six cores with a 1.27-cm diameter and parallel to the muscle fiber were sheared with a Warner-Bratzler head attached to an Instron Universal Testing Machine (model 3343; Instron Corporation, Canton, MA). The Warner-Bratzler head moved at a cross head speed of 200 mm/min. The average peak load (kg) of six cores from the same steak was calculated and the logarithm of WBSF was subsequently analyzed. The weight lost during cooking was recorded and cooking loss was expressed as a percentage of the cooked weight out of the thaw weight.

The second steak was used to measure tenderness, juiciness, connective tissue and flavor by a sensory panel according to the American Meat Science Association Sensory Guidelines [104]. The sensory panel consisted of eight to eleven trained members, and six animals were assessed per session. Two 1 × 2.54 cm samples from each steak were provided to each panelist. Sensory panel measurements analyzed by the sensory panelists included: tenderness (8 = extremely tender, 7 = very tender, 6 = moderately tender, 5 = slightly tender, 4 = slightly tough, 3 = moderately tough, 2 = very tough, 1 = extremely tough), juiciness (8 = extremely juicy, 7 = very juicy, 6 = moderately juicy, 5 = slightly juicy, 4 = slightly dry, 3 = moderately dry, 2 = very dry, 1 = extremely dry), connective tissue (8 = none detected, 7 = practically none, 6 = traces amount, 5 = slight amount, 4 = moderate amount, 3 = slightly abundant, 2 = moderately abundant, 1 = abundant amount), and flavor (8 = extremely intense, 7 = very intense, 6 = moderately intense, 5 = slightly intense, 4 = slightly bland, 3 = moderately bland, 2 = very bland, 1 = extremely bland). For each phenotype, the average of scores from all members of the panel was analyzed for each steak. The Johnson normalization was applied to connective tissue [103].

### Genotyping and data quality control

Genomic DNA was extracted from blood using the DNeasy Blood & Tissue kit (Qiagen, Valencia, CA) and stored at − 20 °C. All animals were genotyped with the commercial GGP Bovine F-250 chip (GeneSeek, Inc., Lincoln, NE) which contains 221,077 single nucleotide polymorphisms (SNPs). A total number of 115,287 SNPs were included in the GWAs after excluding markers with a minor allele frequency lower than 0.05 and a calling rate smaller than 0.9. Samples with calling rates smaller than 0.85 were excluded from the association analysis. All quality control was performed with JMP genomics 6.0 software [103].

### Genome wide association analysis

The analysis was performed using the “Genetics Q-K analysis workflow” of JMP-Genomics 6.0 software applying the mixed model K method [105]. The genomic relationship matrix was calculated and included in the analysis as random effect, and the year of birth and SNP were included as fixed effects. Each marker was tested individually. An adjusted genome wide threshold of 0.6 × 10^− 6^ was calculated by the R function “simpleM_Ex” [106, 107] which applied the effective number of independent tests. The Benjamini-Hochberg *p*-value correction for multiple testing was calculated using the effective number of independent tests. If no SNP reached the genome wide threshold by trait, a secondary arbitrary threshold of 0.1 × 10^− 3^ was used. The R package “CMplot” v3.3.0 [108] was used to graph the p-value distributions.

### Gene enrichment analysis

A tissue restricted gene enrichment was performed for each GWA. The methodology described by Baranzini et al. [109] was modified and carried out using in-house JAVA scripts. From each GWA, all SNPs with p-value ≤0.05 were included in the gene enrichment analysis and were defined as “raw” associated SNPs. Raw associated SNPs were assigned to genes if they were located inside a gene or within 3 kilobases upstream or downstream from a gene. Gene locations were obtain using Biomart from the *Bos taurus* UMD 3.1.1 assembly in Ensembl [110, 111]. The tissue restriction was performed by filtering genes expressed in bovine skeletal muscle, based on a list of 10,919 genes reported by the EMBL-EBI Expression Atlas (Additional file 12). The list included genes reported as expressed in at least one of the two types of experiments with codes E-MTAB-2798 and E-MTAB-2596. This list was also used as a background list in the tissue restricted gene enrichment analysis. The correlation between the raw associated SNPs within each gene was calculated based on their *p*-value, and SNPs were considered correlated when r^2^ > ±0.3. A set of the most significant uncorrelated SNPs for each gene were retained for each gene. When all raw associated SNPs within a gene were correlated, the gene was included only once in the final gene list. Genes with more than one raw associated uncorrelated SNPs were included as many times as the number of uncorrelated SNPs in the final gene list to identify genes with possibly multiple functional polymorphisms. The maximum number of raw associated uncorrelated SNPs for one gene was three (Additional file 13).

The available GO terms for molecular function, cellular component and biological process were included in the analysis using a GO term estimation for the background list in DAVID Bioinformatic Resources 6.8 server [112]. A total number of 105, 89, 85, 92, 81, 86, and 95 GO terms for WBSF, marbling, cooking loss, tenderness, juiciness, connective tissue, and flavor, respectively, were included (Additional file 14). Gene lists with 1714, 1636, 1632, 1753, 1753, 1666, and 1684 genes for WBSF, marbling, cooking loss, tenderness, juiciness, connective tissue, and flavor, respectively, were used (Additional file 14). Gene enrichment was calculated using the hypergeometric test available in the Apache Commons Mathematics Library for JAVA [113]. The Benjamini-Hochberg *p*-value correction was performed for each pathway by trait. The R package “limma” [114] was used to visualize gene overlapping between traits if the same pathway was determined as enriched in numerous traits.

A second analysis was performed using DAVID Bioinformatic Resources 6.8 server [112]. SNPs with p-value ≤0.05 from each GWA analysis were assigned to genes as described in the tissue restricted gene enrichment. All genes were included in the list only once (Additional file 14), regardless of how many uncorrelated associated SNP they contained. A total number of 2762, 2634, 2720, 2788, 2840, 2685, and 2764 genes for WBSF, marbling, cooking loss, tenderness, juiciness, connective tissue, and flavor, respectively, were included in the gene list (Additional file 4). The default DAVID background list was used during the analysis.

### Gene network and candidate genes with multiple QTLs

All gene lists used in the tissue restricted gene enrichment analysis were included in the construction of a gene network represented by a matrix with traits as columns and genes as rows. The weight of each gene for each trait was determined by the number of raw associated uncorrelated SNPs. The R package “igraph” [115] was used to construct the graphical representation of the top 30 most connected genes in the network.

The top five genes in the gene network had a significantly higher connectivity than the rest of the genes in the network and were further analyzed as candidate genes with multiple QTLs associated with different meat quality traits. The LD pattern within each candidate genes with multiple QTLs was predicted using 196 families with 569 steers from the present population. LD blocks were constructed using the Haploview software [116] using a confidence interval of minimum 98% for strong LD [117].

An association analysis was performed for each one of the top five genes in order to confirm the presence of multiple QTLs. All the raw associated uncorrelated SNPs were fitted simultaneously in an association model. The analysis was performed using the “Genetics Q-K analysis workflow” of JMP-Genomics 6.0 software applying the mixed model K method [105] and the final model included only significant SNPs associated with the phenotype that was being considered.

To evaluate the potential effect of missense and synonymous variants identified in these five candidate genes, a bioinformatic analysis was performed. The foldRNA server was used to evaluate the MFE and mRNA structure parameters for each mRNA variant [118]. The Prosite from expasy [119], Phobius [120], TMHMM [121, 122], and SignalP 4.1 [123] servers were used to predict domains, transmembrane regions and signal peptides in each protein, respectively. ComputePI from Expasy [124] was used to calculate the isoelectic point of each protein variant.

### Candidate structural protein assessment of proteolysis

A protease substrate analysis for each of the structural proteins coded by genes identified in the previous analyses was performed. Genes identified through GWA analysis, either one of the gene enrichment procedures and the gene network analysis were included. Genes identified in at least two traits simultaneously for the tissue restricted gene enrichment and in at least three traits for the DAVID functional classification were analyzed. Only genes expressed in bovine muscle or adipose tissue (experiments with codes E-MTAB-2798 and E-MTAB-2596) based on the expression atlas [125] were included in the analysis. This analysis was carried out using the PROSPER server [126] which reports proteins that are possible substrates of μ-calpain.

## Additional files

Additional file 1: GWA results for WBSF, marbling, cooking loss, tenderness, juiciness, connective tissue, and flavor. The phenotypes were measured in *longissimus dorsi* muscle in Brahman-Angus crossbreed steers. SNP location is shown using the Bos_taurus_UMD_3.1.1 assembly. Gene ontology by gene is presented. (XLS 46 kb)

Additional file 2: Enriched clusters across meat quality related phenotypes determined by the DAVID server. The phenotypes were measured in *longissimus dorsi* muscle from an Angus-Brahman crossbred population. Significance of each enriched term was adjusted applying the Benjamini-Hochberg *p*-value correction. (XLS 37 kb)

Additional file 3: Genes identified as enriched by GO term in the tissue restricted gene enrichment analysis. A total number of ten GO terms were identified as enriched. These GO terms were identified for meat quality related traits measured in the *longissimus dorsi* muscle from Brahman-Angus crossbreed steers. Bold gene IDs were accounted twice, provided that they had two associated uncorrelated SNPs. (XLS 47 kb)

Additional file 4: Gene lists and genes identified as enriched using the DAVID based enrichment approach. These genes were found enriched for meat quality related traits recorded in *longissimus dorsi* muscle from Brahman-Angus crossbreed steers. The bolded enriched genes were found in at least four traits simultaneously. (XLS 566 kb)

Additional file 5: Association analysis for ANO2 and NTF3 genes and cooking loss in detail. Cooking loss was measured in the *longissimus dorsi* muscle on 672 Brahman-Angus crossbreed steers. * = p-value distribution fitting each SNP at the time; + = p-value distribution fitting the rs137723969 SNP (arrow) as fixed effect and the remaining SNPs individually. Location of both genes and LD block prediction is presented. Dotted horizontal line is the 0.5*10^− 1^ threshold and black line is the 0.6*10^− 6^ threshold. The SNP correlation heat map for SNPs below the 0.1*10^− 3^ p-value threshold is presented (JPG 2087 kb)

Additional file 6: Title of data: Protease substrate analysis for the uncovered structural proteins. Genes that were uncovered using the genome wide association, the gene enrichment procedures and the gene-trait network analysis were assessed. The PROSPER server was used to identify proteins that are substrates of μ-calpain. (XLS 43 kb)

Additional file 7: Association analysis for EVC2 and WBSF, marbling, cooking loss and taste panel in detail. Phenotypes were measured in the *longissimus dorsi* muscle on Brahman-Angus crossbreed steers. Location of both genes and LD block prediction is presented. Vertical lines highlights the associated uncorrelated SNP by trait. Dotted horizontal line is the 0.5*10^− 1^ threshold and black line is the 0.1*10^− 3^ threshold. The arrows show the SNPs that are required to explain all the variability present in each trait. (JPG 1813 kb)

Additional file 8: Uncorrelated SNPs in *EVC2*, *ANXA10*, *PALLD*, *PKHD1* and *ENSBTAG00000020151* genes simultaneously associated with meat quality. (XLS 43 kb)

Additional file 9: Association analysis for ANXA10 and PALLD and WBSF, marbling, cooking loss and taste panel in detail. Phenotypes were measured in the *longissimus dorsi* muscle on Brahman-Angus crossbreed steers. Location of both genes and LD block prediction is presented. Vertical lines highlights the associated uncorrelated SNP by trait. Dotted horizontal line is the 0.5*10^− 1^ threshold and black line is the 0.1*10^− 3^ threshold. The arrows show the SNPs that are required to explain all the variability present in each trait. (JPG 7606 kb)

Additional file 10: Association analysis for PKHD1 and WBSF, marbling, cooking loss and taste panel in detail. Phenotypes were measured in the *longissimus dorsi* muscle on Brahman-Angus crossbreed steers. Location of both genes and LD block prediction is presented. Vertical lines highlight the associated uncorrelated SNP by trait. Dotted horizontal line is the 0.5*10^− 1^ threshold and black line is the 0.1*10^− 3^ threshold. The arrows show the SNPs that are required to explain all the variability present in each trait (JPG 2876 kb)

Additional file 11: Association analysis for ENSBTAG00000020151 and WBSF, marbling, cooking loss and taste panel in detail. Phenotypes were measured in the *longissimus dorsi* muscle on Brahman-Angus crossbreed steers. Location of both genes and LD block prediction is presented. Vertical lines highlights the associated uncorrelated SNP by trait. Dotted horizontal line is the 0.5*10^− 1^ threshold and black line is the 0.1*10^− 3^ threshold. The arrows show SNPs that could be fitted simultaneously in the association model by each trait. The arrows show the SNPs that are required to explain all the variability present in each trait. (JPG 1750 kb)

Additional file 12: List with 10,919 genes reported as expressed in bovine skeletal muscle by the EMBL-EBI Expression Atlas. A gene was considered as expressed in bovine skeletal muscle if it was present in E-MTAB-2798 or E-MTAB-2596 assays. This list was used as background list for the tissue restricted gene enrichment analysis. (XLS 875 kb)

Additional file 13: Genes identified by the GWA analysis and included in the final gene lists by trait. These gene lists were used for the gene enrichment analysis and the gene-trait network construction. It is presented the frequency of genes with one or multiple associated uncorrelated SNPs (JPG 69 kb)

Additional file 14: List of GO terms and gene lists included in the tissue restricted gene enrichment analysis. A total number of 105, 89, 85, 92, 81, 86, and 95 GO terms for WBSF, marbling, cooking loss, tenderness, juiciness, connective tissue, and flavor respectively, were included in the analysis. These sets of GO terms were analyzed provided a GO term prediction analysis for the list used as background with DAVID Bioinformatic Resources 6.8 server [112]. The gene lists used in this analysis show the stable gene ID from ensembl and the number of associated uncorrelated SNPs of each gene by trait. (XLS 534 kb)

## Abbreviations

BTA: *Bos taurus* autosome
cAMP: Cyclic adenosine 3′:5′-monophosphate
ER: Endoplasmic reticulum
GDP: Guanosine-5′-diphosphate
GO: Gene ontology
GTP: Guanosine-5′-triphosphate
GWA: Genome wide association
LD: Linkage disequilibrium
MFE: Minimum Free Energy
QTL: Quantitative trait loci
SNPs: Single nucleotide polymorphisms
WBSF: Warner-Bratzler Shear Force

## References

[1] Calkins CR, Hodgen JM (2007). A fresh look at meat flavor. Meat Sci.

[2] Hocquette JF, Van Wezemael L, Chriki S, Legrand I, Verbeke W, Farmer L (2014). Modelling of beef sensory quality for a better prediction of palatability. Meat Sci.

[3] Mateescu RG (2015). Genetics of meat quality. The genetics of cattle.

[4] Renand G, Picard B, Touraille C, Berge P, Lepetit J (2001). Relationships between muscle characteristics and meat quality traits of young Charolais bulls. Meat Sci.

[5] Riley JM, Schroeder TC, Wheeler TL, Shackelford SD, Koohmaraie M. Valuing Fed Cattle Using Objective Tenderness Measures. J Agric Appl Econ. 2009;(1):163–75.

[6] Mateescu RG, Oltenacu PA, Garmyn AJ, Mafi GG, VanOverbeke DL (2016). Strategies to predict and improve eating quality of cooked beef using carcass and meat composition traits in Angus cattle. J Anim Sci.

[7] Houle D, Govindaraju DR, Omholt S (2010). Phenomics: the next challenge. Nat Rev Genet.

[8] McClure MC, Ramey HR, Rolf MM, McKay SD, Decker JE, Chapple RH (2012). Genome-wide association analysis for quantitative trait loci influencing Warner-Bratzler shear force in five taurine cattle breeds. Anim Genet.

[9] Bush WS, Moore JH. Chapter 11: genome-wide association studies. PLoS Comput Biol. 2012;8. 10.1371/journal.pcbi.1002822 PMC3531285 23300413

[10] Elzo MA, Johnson DD, Wasdin JG, Driver JD (2012). Carcass and meat palatability breed differences and heterosis effects in an Angus-Brahman multibreed population. Meat Sci.

[11] Elzo MA, Mateescu RG, Rae DO, Carr CC, Scheffler TL, Scheffler JM (2018). Genomic-polygenic EBV for reproduction , ultrasound-carcass , and tenderness traits in the Florida multibreed Brahman-Angus population. Proceedings of the World Congress on Genetics Applied to Livestock Production.

[12] Shackelford SD, Da K, Wheeler TL, Meadows LR, MEO C (2012). National Beef Quality Audit – 2011 : Survey of instrument grading assessments of.

[13] Elzo MA, Mateescu R, Thomas MG, Johnson DD, Martinez CA, Rae DO (2016). Growth and reproduction genomic-polygenic and polygenic parameters and prediction trends as Brahman fraction increases in an Angus-Brahman multibreed population. Livest Sci.

[14] Elzo MA, Thomas MG, Martinez CA, Lamb GC, Johnson DD, Rae DO (2014). Genomic-polygenic evaluation of multibreed Angus-Brahman cattle for postweaning feed efficiency and growth using actual and imputed Illumina50k SNP genotypes. Livest Sci.

[15] Karasik D, Kiel DP (2008). Genetics of the musculoskeletal system: a pleiotropic approach. J Bone Miner Res.

[16] Kawao N, Kaji H (2015). Interactions between muscle tissues and bone metabolism. J Cell Biochem.

[17] Chen L, Ekine-Dzivenu C, Vinsky M, Basarab J, Aalhus J, Dugan MER (2015). Genome-wide association and genomic prediction of breeding values for fatty acid composition in subcutaneous adipose and longissimus lumborum muscle of beef cattle. BMC Genet.

[18] Mateescu RG, Garrick DJ, Reecy JM. Network analysis reveals putative genes affecting meat quality in Angus cattle. Front Genet. 2017;8 NOV. 10.3389/fgene.2017.00171 PMC5681485 29163638

[19] Ramayo-Caldas Y, Renand G, Ballester M, Saintilan R, Rocha D (2016). Multi-breed and multi-trait co-association analysis of meat tenderness and other meat quality traits in three French beef cattle breeds. Genet Sel Evol.

[20] Gill JL, Bishop SC, McCorquodale C, Williams JL, Wiener P (2009). Association of selected SNP with carcass and taste panel assessed meat quality traits in a commercial population of Aberdeen Angus-sired beef cattle. Genet Sel Evol.

[21] Tizioto PC, Decker JE, Taylor JF, Schnabel RD (2013). Mudadu M a, Silva FL, et al. genome scan for meat quality traits in Nelore beef cattle. Physiol Genomics.

[22] Leal-Gutiérrez JD, Elzo MA, Johnson DD, Scheffler TL, Scheffler JM, Mateescu RG. Association of μ-Calpain and Calpastatin Polymorphisms with Meat Tenderness in a Brahman–Angus Population. Front Genet. 2018;9 February:1–10. doi:10.3389/fgene.2018.00056. 10.3389/fgene.2018.00056 PMC5827160 29520298

[23] Wright SA, Ramos P, Johnson DD, Scheffler JM, Elzo MA, Mateescu RG, et al. Brahman genetics influence muscle fiber properties, protein degradation, and tenderness in an Angus-Brahman multibreed herd. Meat Sci. 2018;135 November:84–93. 10.1016/j.meatsci.2017.09.006 28946054

[24] Chiarelli N, Carini G, Zoppi N, Ritelli M, Colombi M (2018). Transcriptome analysis of skin fibroblasts with dominant negative COL3A1 mutations provides molecular insights into the etiopathology of vascular Ehlers-Danlos syndrome. PLoS One.

[25] Uezumi A, Ito T, Morikawa D, Shimizu N, Yoneda T, Segawa M (2011). Fibrosis and adipogenesis originate from a common mesenchymal progenitor in skeletal muscle. J Cell Sci.

[26] Chen SL, Dowhan DH, Hosking BM, Muscat GEO (2000). The steroid receptor coactivator, GRIP-1, is necessary for MEF-2C-dependent gene expression and skeletal muscle differentiation. Genes Dev.

[27] Sartorelli V, Caretti G. Mechanisms underlying the transcriptional regulation of skeletal myogenesis. Curr Opin Genet Dev. 2005;15 5 SPEC. ISS.:528–35. 10.1016/j.gde.2005.04.015 PMC1283108 16055324

[28] Wannenes F, Caprio M, Gatta L, Fabbri A, Bonini S, Moretti C (2008). Androgen receptor expression during C2C12 skeletal muscle cell line differentiation. Mol Cell Endocrinol.

[29] Liu H, Yan P, Fanning E (2015). Human DNA helicase B functions in cellular homologous recombination and stimulates rad51-mediated 5′ -3′ heteroduplex extension in vitro. PLoS One.

[30] Cesar AS, Regitano LC, Mourão GB, Tullio RR, Lanna DP, Nassu RT (2014). Genome-wide association study for intramuscular fat deposition and composition in Nellore cattle. BMC Genet.

[31] Islam MN, Fox D, Guo R, Enomoto T, Wang W (2010). RecQL5 promotes genome stabilization through two parallel mechanisms--interacting with RNA polymerase II and acting as a helicase. Mol Cell Biol.

[32] Neureither F, Ziegler K, Pitzer C, Frings S, Möhrlen F (2017). Impaired motor coordination and learning in mice lacking Anoctamin 2 calcium-gated chloride channels. Cerebellum.

[33] Chevrel G, Hohlfeld R, Sendtner M (2006). The role of neurotrophins in muscle under physiological and pathological conditions. Muscle Nerve.

[34] Sheard PW, Bewick GS, Woolley AG, Shaw J, Fisher L, Fong SW (2010). Investigation of neuromuscular abnormalities in neurotrophin-3-deficient mice. Eur J Neurosci.

[35] Tran TTT, Poirier H, Clément L, Nassir F, Pelsers MMAL, Petit V (2011). Luminal lipid regulates CD36 levels and downstream signaling to stimulate chylomicron synthesis. J Biol Chem.

[36] Bonen A, Parolin M, Steinberg G, Calles-Escandon J, Tandon N, Glatz J (2004). Triacylglycerol accumulation in human obesity and type 2 diabetes is associated with increased rates of skeletal muscle fatty acid transport and increased sarcolemmal FAT/CD36. FASEB J.

[37] Dang CG, Cho SH, Sharma A, Kim HC, Jeon GJ, Yeon SH (2014). Genome-wide association study for warner-bratzler shear force and sensory traits in Hanwoo (Korean cattle). Asian-Australasian J Anim Sci.

[38] Urano T, Shiraki M, Yagi H, Ito M, Sasaki N, Sato M (2012). GPR98 / Gpr98 gene is involved in the regulation of human and mouse bone mineral density. J Clin Endocrinol Metab.

[39] Mellman DL, Gonzales ML, Song C, Barlow CA, Wang P, Kendziorski C (2008). A PtdIns4,5P2-regulated nuclear poly(a) polymerase controls expression of select mRNAs. Nature.

[40] Lorenzon E, Colladel R, Andreuzzi E, Marastoni S, Todaro F, Schiappacassi M (2012). MULTIMERIN2 impairs tumor angiogenesis and growth by interfering with VEGF-A/VEGFR2 pathway. Oncogene.

[41] Castro LM, Rosa GJM, Lopes FB, Regitano LCA, Rosa AJM, Magnabosco CU. Genomewide association mapping and pathway analysis of meat tenderness in polled Nellore cattle. J Anim Sci. 2017;95. 10.2527/jas.2016.1348 28727016

[42] Tsai L, Schwake M, Corbett M, Gecz Y, Berkovic S (2013). GOSR 2: a novel form of congenital muscular dystrophy. Neuromuscul Disord.

[43] van Egmond ME, Verschuuren-Bemelmans CC, Nibbeling EA, Elting JWJ, Sival DA, Brouwer OF (2014). Ramsay hunt syndrome: clinical characterization of progressive myoclonus ataxia caused by GOSR2 mutation. Mov Disord.

[44] Uemoto Y, Abe T, Tameoka N, Hasebe H, Inoue K, Nakajima H (2011). Whole-genome association study for fatty acid composition of oleic acid in Japanese black cattle. Anim Genet.

[45] Zheng X, Dumitru R, Lackford BL, Freudenberg JM, Singh AP, Archer TK (2012). Cnot1, Cnot2, and Cnot3 maintain mouse and human ESC identity and inhibit extraembryonic differentiation. Stemm dells.

[46] Morita M, Oike Y, Nagashima T, Kadomatsu T, Tabata M, Suzuki T (2011). Obesity resistance and increased hepatic expression of catabolism-related mRNAs in Cnot3 +/− mice. EMBO J.

[47] Ndongo-Thiam N, De Sallmard G, Kastrup J, Miossec P (2014). Levels of soluble osteoclast-associated receptor (sOSCAR) in rheumatoid arthritis: link to disease severity and cardiovascular risk. Ann Rheum Dis.

[48] Saatchi M, Garrick DJ, Tait RG, Mayes MS, Drewnoski M, Schoonmaker J (2013). Genome-wide association and prediction of direct genomic breeding values for composition of fatty acids in Angus beef cattle. BMC Genomics.

[49] Li N, Li Y, Lv J, Zheng X, Wen H, Shen H (2016). ZMYND8 reads the dual histone mark H3K4me1-H3K14ac to antagonize the expression of metastasis-linked genes. Mol Cell.

[50] Bhattacharyya D, Glick BS (2007). Two mammalian Sec16 homologues have nonredundant functions in endoplasmic reticulum (ER) export and transitional ER organization. Mol Biol Cell.

[51] Park SH, Zhu P-P, Parker RL, Blackstone C. Hereditary spastic paraplegia proteins REEP1, spastin, and atlastin-1 coordinate microtubule interactions with the tubular ER network. J Clin Invest. 2010;120. 10.1172/JCI40979 PMC2846052 20200447

[52] Boscia F, D’Avanzo C, Pannaccione A, Secondo A, Casamassa A, Formisano L (2012). Silencing or knocking out the Na+/Ca2+exchanger-3 (NCX3) impairs oligodendrocyte differentiation. Cell Death Differ.

[53] Doyle C, Strominger JL (1987). Interaction between CD4 and class II MHC molecules mediates cell adhesion. Nature.

[54] Beetz C, Koch N, Khundadze M, Zimmer G, Nietzsche S, Hertel N (2014). A spastic paraplegia mouse model reveals REEP1-dependent ER shaping. J Clin Invest.

[55] Boehm ML, Kendall TL, Thompson VF, Goll DE, Boehm ML, Kendall TL (1998). Changes in the calpains and calpastatin during postmortem storage of bovine muscle. J Anim Sci.

[56] Geesink GH, Kuchay S, Chishti AH, Koohmaraie M (2006). μ-Calpain is essential for postmortem proteolysis of muscle proteins. J Anim Sci.

[57] Lametsch R, Roepstorff P, Møller HS, Bendixen E (2004). Identification of myofibrillar substrates for μ-calpain. Meat Sci.

[58] Lomiwes D, Farouk MM, Wu G, Young OA (2014). The development of meat tenderness is likely to be compartmentalised by ultimate pH. Meat Sci.

[59] Wang J, Qin S, Li F, Li S, Zhang W, Peng J (2014). Crystal structure of human BS69 Bromo-ZnF-PWWP reveals its role in H3K36me3 nucleosome binding. Cell Res.

[60] Monterisi S, Lobo MJ, Livie C, Castle JC, Weinberger M, Baillie G (2017). PDE2A2 regulates mitochondria morphology and apoptotic cell death via local modulation of cAMP/PKA signalling. elife.

[61] Maloverjan A, Piirsoo M, Michelson P, Kogerman P, Østerlund T (2010). Identification of a novel serine/threonine kinase ULK3 as a positive regulator of hedgehog pathway. Exp Cell Res.

[62] Alves C, Sunyach C, Giaime E, West A, Corti O, Brice A (2010). Transcriptional repression of p53 by parkin and impairment by mutations associated with autosomal recessive juvenile Parkinson’s disease. October.

[63] Attanasio M, Uhlenhaut NH, Sousa VH, O’Toole JF, Otto E, Anlag K (2007). Loss of GLIS2 causes nephronophthisis in humans and mice by increased apoptosis and fibrosis. Nat Genet.

[64] Khanna N, Ge Y, Chen J. MicroRNA-146b promotes myogenic differentiation and modulates multiple gene targets in muscle cells. PLoS One. 2014;9. 10.1371/journal.pone.0100657 PMC4067360 24956113

[65] Li Z, Gilbert JA, Zhang Y, Zhang M, Qiu Q, Ramanujan K (2012). An HMGA2-IGF2BP2 Axis regulates myoblast proliferation and Myogenesis. Dev Cell.

[66] Kim KS, Thomsen H, Bastiaanse J, Thu Nguyen N, Dekkers JCM, Plastow GS (2004). Investigation of obesity candidate genes on porcine fat deposition quantitative trait loci regions. Obes Res.

[67] Deng J, Lewis PA, Greggio E, Sluch E, Beilina A, Cookson MR (2008). Structure of the ROC domain from the Parkinson’s disease-associated leucine-rich repeat kinase 2 reveals a dimeric GTPase. Proc Natl Acad Sci.

[68] Raphael AR, Couthouis J, Sakamuri S, Siskind C, Vogel H, Day JW (2014). Congenital muscular dystrophy and generalized epilepsy caused by GMPPB mutations. Brain Res.

[69] Luo S, Cai S, Maxwell S, Yue D, Zhu W, Qiao K (2017). Novel mutations in the C-terminal region of GMPPB causing limb-girdle muscular dystrophy overlapping with congenital myasthenic syndrome. Neuromuscul Disord.

[70] Magalhães AFB, de Camargo GMF, Fernandes GA, Gordo DGM, Tonussi RL, Costa RB (2016). Genome-wide association study of meat quality traits in Nellore cattle. PLoS One.

[71] Nakanishi H, Nakamura T, Canaani E, Croce CM (2007). ALL1 fusion proteins induce deregulation of EphA7 and ERK phosphorylation in human acute leukemias. Proc Natl Acad Sci U S A.

[72] Vickerman L, Neufeld S, Cobb J (2011). Shox2 function couples neural, muscular and skeletal development in the proximal forelimb. Dev Biol.

[73] Lai KO, Ip FCF, Cheung J, Fu AKY, Ip NY (2001). Expression of Eph receptors in skeletal muscle and their localization at the neuromuscular junction. Mol Cell Neurosci.

[74] See K, Yadav P, Giegerich M, Cheong PS, Graf M, Vyas H (2014). SMN deficiency alters Nrxn2 expression and splicing in zebrafish and mouse models of spinal muscular atrophy. Hum Mol Genet.

[75] Ruiz-Perez VL, Blair HJ, Rodriguez-Andres ME, Blanco MJ, Wilson A, Liu Y-N (2007). Evc is a positive mediator of Ihh-regulated bone growth that localises at the base of chondrocyte cilia. Development.

[76] Tompson SWJ, Ruiz-Perez VL, Blair HJ, Barton S, Navarro V, Robson JL (2007). Sequencing EVC and EVC2 identifies mutations in two-thirds of Ellis-van Creveld syndrome patients. Hum Genet.

[77] Rescher U, Gerke V (2004). Annexins - unique membrane binding proteins with diverse functions. J Cell Sci.

[78] Dixon RDS, Arneman DK, Rachlin AS, Sundaresan NR, Costello MJ, Campbell SL (2008). Palladin is an actin cross-linking protein that uses immunoglobulin-like domains to bind filamentous actin. J Biol Chem.

[79] Gunay-Aygun M, Tuchman M, Font-Montgomery E, Lukose L, Edwards H, Garcia A (2010). PKHD1 sequence variations in 78 children and adults with autosomal recessive polycystic kidney disease and congenital hepatic fibrosis. Mol Genet Metab.

[80] Duan J, Huang H, Lv X, Wang H, Tang Z, Sun H, et al. PKHD1 post-transcriptionally modulated by miR-365-1 inhibits cell-cell adhesion. 2012; March:382–9. 10.1002/cbf.2795 22411058

[81] Ito Y, Sekine A, Takada D, Yabuuchi J, Kogure Y, Ueno T (2017). Renal histology and MRI findings in a 37-year-old Japanese patient with autosomal recessive polycystic kidney disease. Clin Nephrol.

[82] Nagasawa Y, Matthiesen S, Onuchic LF, Hou X, Bergmann C, Esquivel E (2002). Identification and characterization of Pkhd1, the mouse orthologue of the human ARPKD gene. J Am Soc Nephrol.

[83] Zerbino DR, Achuthan P, Akanni W, Amode MR, Barrell D, Bhai J (2018). Ensembl 2018. Nucleic Acids Res.

[84] Posey AD, Swanson KE, Alvarez MG, Krishnan S, Earley JU, Band H (2014). EHD1 mediates vesicle trafficking required for normal muscle growth and transverse tubule development. Dev Biol.

[85] Guilherme A, Soriano NA, Furcinitti PS, Czech MP (2004). Role of EHD1 and EHBP1 in perinuclear sorting and insulin-regulated GLUT4 recycling in 3T3-L1 adipocytes. J Biol Chem.

[86] Jovic M, Naslavsky N, Rapaport D, Horowitz M, Caplan S (2007). EHD1 regulates beta1 integrin endosomal transport: effects on focal adhesions, cell spreading and migration. J Cell Sci.

[87] Granucci F, Petralia F, Urbano M, Citterio S, Di Tota F, Ricciardi-castagnoli P (2012). The scavenger receptor MARCO mediates cytoskeleton rearrangements in dendritic cells and microglia the scavenger receptor MARCO mediates cytoskeleton rearrangements in dendritic cells and microglia. October.

[88] Maes H, Van Eygen S, Krysko DV, Vandenabeele P, Nys K, Rillaerts K (2014). BNIP3 supports melanoma cell migration and vasculogenic mimicry by orchestrating the actin cytoskeleton. Cell Death Dis.

[89] Zoppi N, Gardella R, De Paepe A, Barlati S, Colombi M (2004). Human fibroblasts with mutations in COL5A1 and COL3A1 genes do not organize collagens and fibronectin in the extracellular matrix, Down-regulate α2β1Integrin, and recruit αvβ3Instead of α5β1Integrin. J Biol Chem.

[90] Yadav R, Rimerman R, Scofield MA, Dravid SM (2011). Mutations in the transmembrane domain M3 generate spontaneously open orphan glutamate delta 1 receptor. Brain Res.

[91] Venco P, Bonora M, Giorgi C, Papaleo E, Iuso A, Prokisch H, et al. Mutations of C19orf12, coding for a transmembrane glycine zipper containing mitochondrial protein, cause mis-localization of the protein, inability to respond to oxidative stress and increased mitochondrial Ca2+. Front Genet. 2015;6 MAY:1–14. 10.3389/fgene.2015.00185 PMC4470416 26136767

[92] Velghe AI, Van Cauwenberghe S, Polyansky AA, Chand D, Montano-Almendras CP, Charni S (2014). PDGFRA alterations in cancer: characterization of a gain-of-function V536E transmembrane mutant as well as loss-of-function and passenger mutations. Oncogene.

[93] Cho S, Kang SM, Seong P, Kang G, Kim Y, Kim J (2016). Effect of aging time on physicochemical meat quality and sensory property of Hanwoo bull beef. Korean J food Sci Anim Resour.

[94] Koohmaraie M, Geesink GH (2006). Contribution of postmortem muscle biochemistry to the delivery of consistent meat quality with particular focus on the calpain system. Meat Sci.

[95] Bee G, Anderson AL, Lonergan SM, Huff-Lonergan E (2007). Rate and extent of pH decline affect proteolysis of cytoskeletal proteins and water-holding capacity in pork. Meat Sci.

[96] Monsón F, Sañudo C, Sierra I (2005). Influence of breed and ageing time on the sensory meat quality and consumer acceptability in intensively reared beef. Meat Sci.

[97] Averna M, Stifanese R, Grosso R, Pedrazzi M, De Tullio R, Salamino F (2010). Role of calpain in the regulation of CFTR (cystic fibrosis transmembrane conductance regulator) turnover. Biochem J.

[98] Bi X, Chang V, Molnar E, McIlhinney RAJ, Baudry M (1996). The C-terminal domain of glutamate receptor subunit 1 is a target for calpain-mediated proteolysis. Neuroscience.

[99] Bi X, Chen J, Dang S, Wenthold RJ, Tocco G, Baudry M (1997). Characterization of calpain-mediated proteolysis of GluR1 subunits of alpha-amino-3-hydroxy-5-methylisoxazole-4-propionate receptors in rat brain. J Neurochem.

[100] Guttmann RP, Sokol S, Baker DL, Simpkins KL, Dong Y, Lynch DR (2002). Proteolysis of the N-methyl-D-aspartate receptor by Calpain in situ. J Pharmacol Exp Ther.

[101] Kopil CM, Vais H, Cheung KH, Siebert AP, Mak DOD, Foskett JK (2011). Calpain-cleaved type 1 inositol 1,4,5-trisphosphate receptor (InsP 3R1) has InsP 3-independent gating and disrupts intracellular ca 2+ homeostasis. J Biol Chem.

[102] Elzo MA, Thomas MG, Johnson DD, Martinez CA, Lamb GC, Rae DO (2015). Genomic-polygenic evaluation of multibreed Angus-Brahman cattle for postweaning ultrasound and weight traits with actual and imputed Illumina50k SNP genotypes. Livest Sci.

[103] JMP®, Version 13. SAS Institute Inc., Cary, NC, 1989–2007.

[104] Belk KE, Dikeman ME, Calkins CR (2015). Andy King D.

[105] Stich B, Möhring J, Piepho HP, Heckenberger M, Buckler ES, Melchinger AE (2008). Comparison of mixed-model approaches for association mapping. Genetics.

[106] Gao X, Starmer J, Martin ER (2008). A multiple testing correction method for genetic association studies using correlated single nucleotide polymorphisms. Genet Epidemiol.

[107] Gao X, Becker LC, Becker DM, Starmer JD, Province M (2010). Avoiding the high Bonferroni penalty in genome-wide association studies. Genet Epidemiol.

[108] LiLin-Yin. CMplot: Circle Manhattan Plot. 2017.

[109] Baranzini SE, Galwey NW, Wang J, Khankhanian P, Lindberg R, Pelletier D (2009). Pathway and network-based analysis of genome-wide association studies in multiple sclerosis. Hum Mol Genet.

[110] Huang DW, Sherman BT, Lempicki RA (2009). Systematic and integrative analysis of large gene lists using DAVID bioinformatics resources. Nat Protoc.

[111] Huang DW, Sherman BT, Lempicki RA (2009). Bioinformatics enrichment tools: paths toward the comprehensive functional analysis of large gene lists. Nucleic Acids Res.

[112] david.ncifcrf.gov. [Internet]. Available from: david.ncifcrf.gov.

[113] commons.apache.org.

[114] Ritchie ME, Phipson B, Wu D, Hu Y, Law CW, Shi W, et al. limma powers differential expression analyses for RNA-sequencing and microarray studies. Nucleic Acids Res. 2015;43:e47. 10.1093/nar/gkv007 PMC4402510 25605792

[115] Csárdi G, Nepusz T (1695). The igraph software package for complex network research. InterJournal Complex Syst.

[116] Barrett JC, Fry B, Maller J, Daly MJ (2005). Haploview: analysis and visualization of LD and haplotype maps. Bioinformatics.

[117] Gabriel SB, Schaffner SF, Nguyen H, Moore JM, Roy J, Blumenstiel B, et al. The structure of haplotype blocks in the human genome. Science (80- ). 2002;296:2225–9. doi:10.1126/science.1069424. 10.1126/science.1069424 12029063

[118] Zuker M. Mfold web server for nucleic acid folding and hybridization prediction. 2003;31:3406–15. 10.1093/nar/gkg595 PMC169194 12824337

[119] de Castro E, Sigrist CJA, Gattiker A, Bulliard V, Langendijk-Genevaux PS, Gasteiger E, et al. ScanProsite: Detection of PROSITE signature matches and ProRule-associated functional and structural residues in proteins. Nucleic Acids Res. 2006;34 WEB. SERV. ISS.:362–5. 10.1093/nar/gkl124 PMC1538847 16845026

[120] Käll L, Krogh A, Sonnhammer ELL (2004). A combined transmembrane topology and signal peptide prediction method. J Mol Biol.

[121] Krogh A, Larsson È, Von Heijne G, Sonnhammer ELL (2001). Predicting transmembrane protein topology with a hidden Markov Model : application to complete genomes.

[122] Sonnhammer E, von Heijne G, Krogh A, Glasgow J, Littlejohn T, Major F, Lathrop R, Sankoff D, Sensen C (1998). A hidden Markov model for predicting transmembrane helices in protein sequences. Sixth international conference on intelligent Systems for Molecular Biology.

[123] Petersen TN, Brunak S, Von Heijne G, Nielsen H (2011). SignalP 4.0: discriminating signal peptides from transmembrane regions. Nat Methods.

[124] Gasteiger E, Gattiker A, Hoogland C, Ivanyi I, Appel RD, Bairoch A (2003). ExPASy: the proteomics server for in-depth protein knowledge and analysis. Nucleic Acids Res.

[125] www.ebi.ac.uk. www.ebi.ac.uk/gxa/home. [Internet]. Available from: www.ebi.ac.uk/gxa/home.

[126] Song J, Tan H, Perry AJ, Akutsu T, Webb GI, Whisstock JC, et al. PROSPER: an integrated feature-based tool for predicting protease substrate cleavage sites. PLoS One. 2012;7. 10.1371/journal.pone.0050300 PMC3510211 23209700

