# Supplementary figures and images for: Genome wide association and gene enrichment analysis reveal membrane anchoring and structural proteins associated with meat quality in beef
Source: BMC Genomics. 2019 Feb 21;20:151. doi: 10.1186/s12864-019-5518-3 (PMC6385435; doi:10.1186/s12864-019-5518-3)

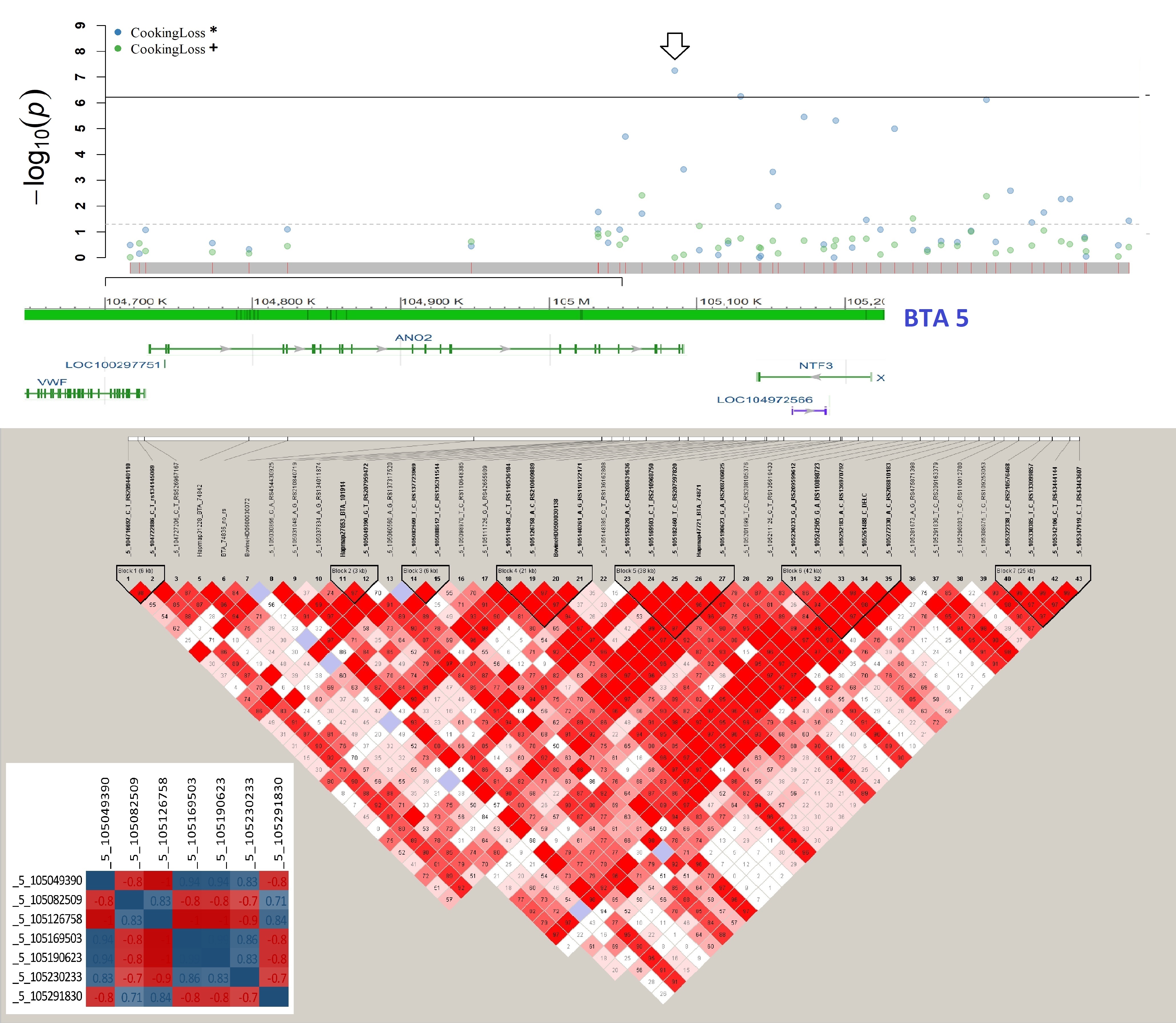

Supplement: Supplementary file 5 — Association analysis for ANO2 and NTF3 genes and cooking loss in detail. Cooking loss was measured in the longissimus dorsi muscle on 672 Brahman-Angus crossbreed steers. * = p-value distribution fitting each SNP at the time; + = p-value distribution fitting the rs137723969 SNP (arrow) as fixed effect and the remaining SNPs individually. Location of both genes and LD block prediction is presented. Dotted horizontal line is the 0.5*10− 1 threshold and black line is the 0.6*10− 6 threshold. The SNP correlation heat map for SNPs below the 0.1*10− 3 p-value threshold is presented (JPG 2087 kb) [file 12864_2019_5518_MOESM5_ESM.jpg]

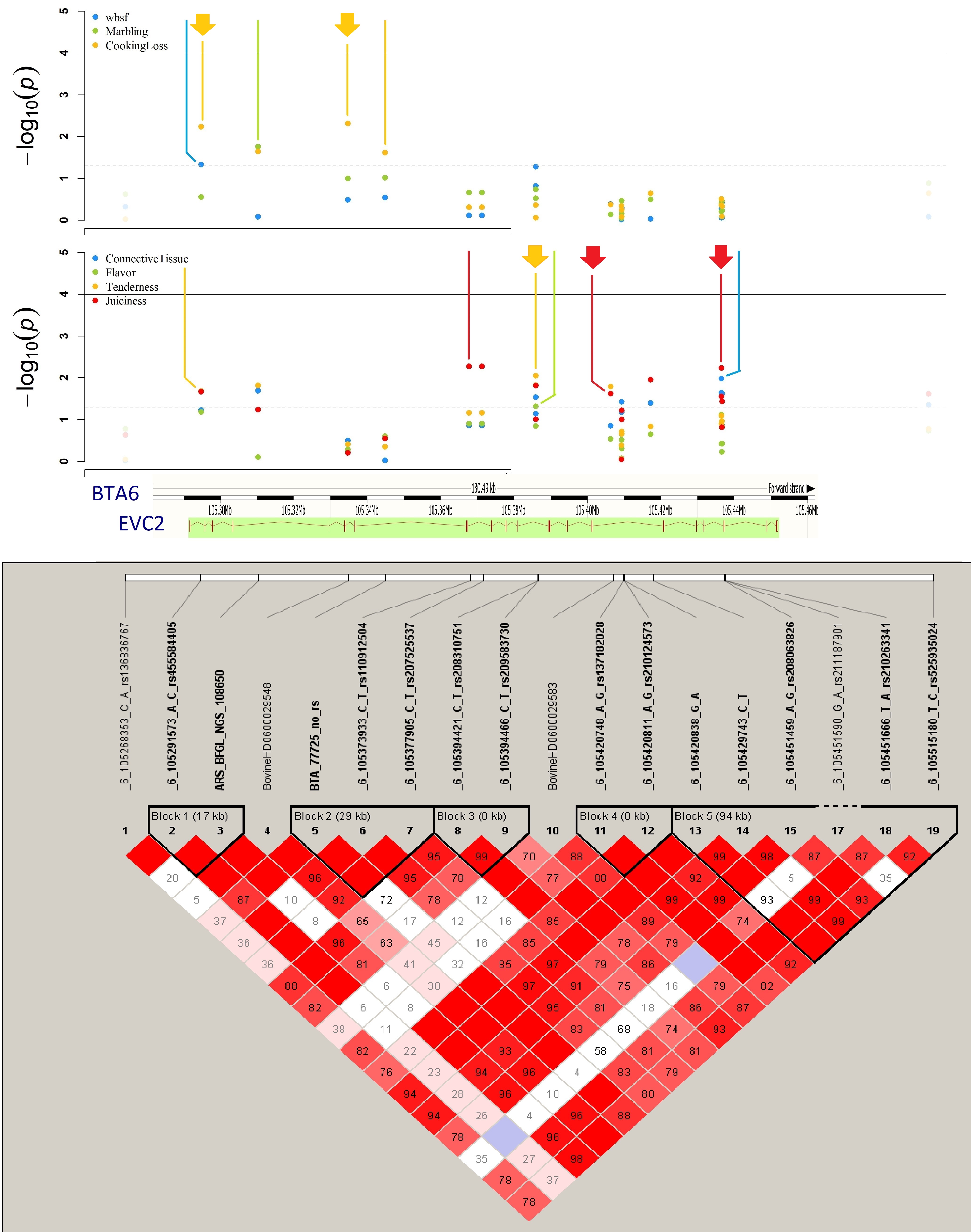

Supplement: Supplementary file 7 — Association analysis for EVC2 and WBSF, marbling, cooking loss and taste panel in detail. Phenotypes were measured in the longissimus dorsi muscle on Brahman-Angus crossbreed steers. Location of both genes and LD block prediction is presented. Vertical lines highlights the associated uncorrelated SNP by trait. Dotted horizontal line is the 0.5*10− 1 threshold and black line is the 0.1*10− 3 threshold. The arrows show the SNPs that are required to explain all the variability present in each trait. (JPG 1813 kb) [file 12864_2019_5518_MOESM7_ESM.jpg]

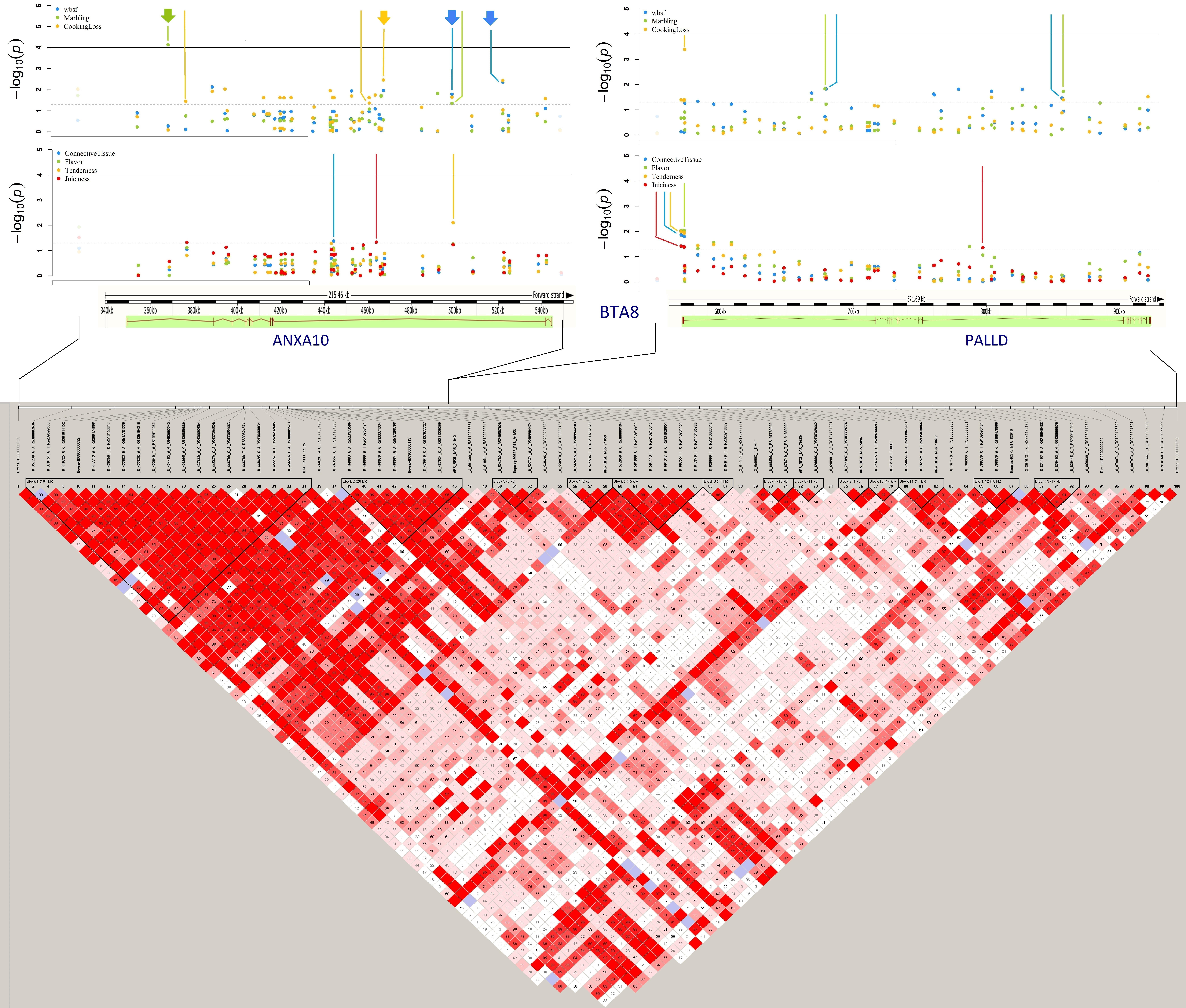

Supplement: Supplementary file 9 — Association analysis for ANXA10 and PALLD and WBSF, marbling, cooking loss and taste panel in detail. Phenotypes were measured in the longissimus dorsi muscle on Brahman-Angus crossbreed steers. Location of both genes and LD block prediction is presented. Vertical lines highlights the associated uncorrelated SNP by trait. Dotted horizontal line is the 0.5*10− 1 threshold and black line is the 0.1*10− 3 threshold. The arrows show the SNPs that are required to explain all the variability present in each trait. (JPG 7606 kb) [file 12864_2019_5518_MOESM9_ESM.jpg]

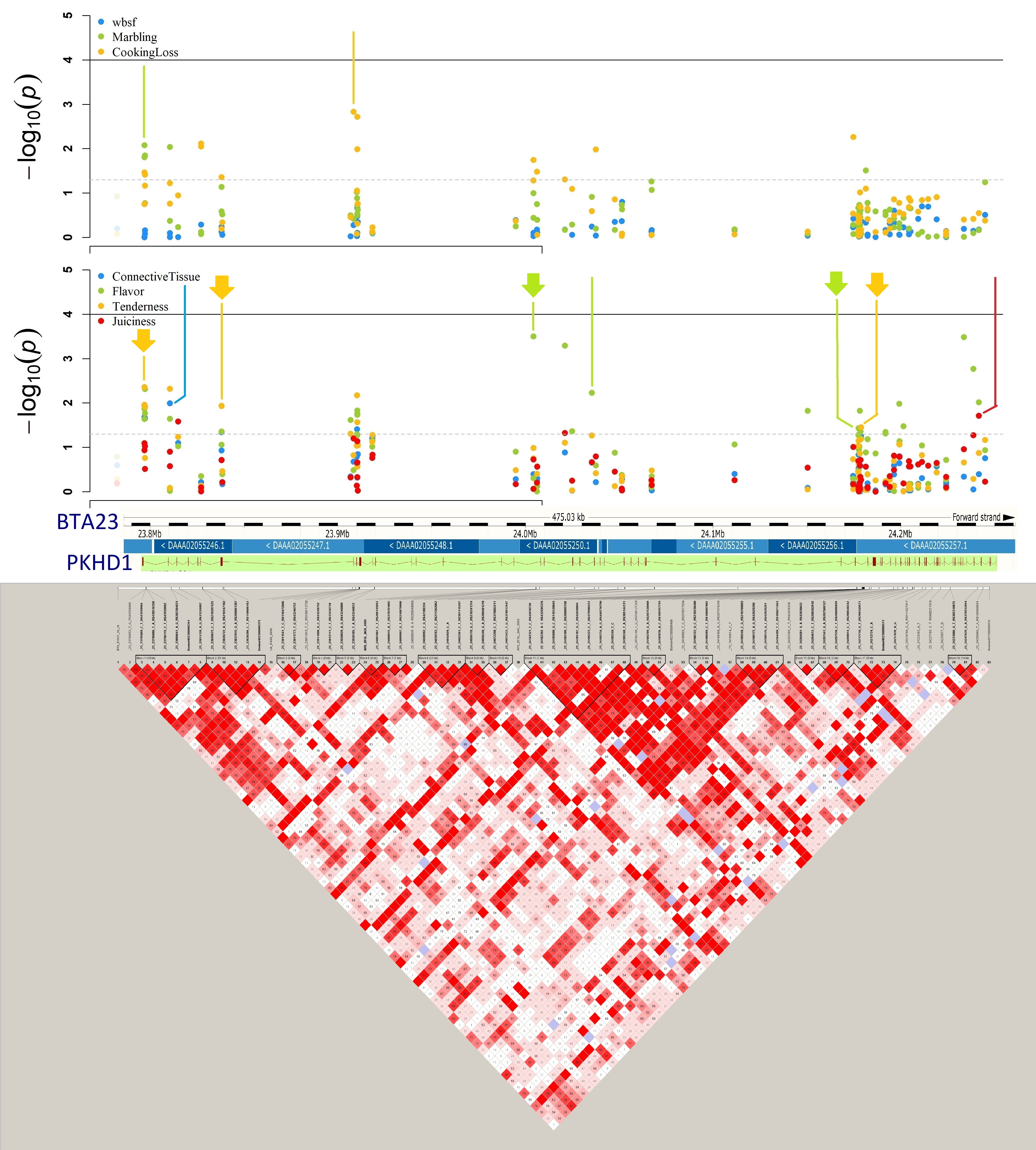

Supplement: Supplementary file 10 — Association analysis for PKHD1 and WBSF, marbling, cooking loss and taste panel in detail. Phenotypes were measured in the longissimus dorsi muscle on Brahman-Angus crossbreed steers. Location of both genes and LD block prediction is presented. Vertical lines highlight the associated uncorrelated SNP by trait. Dotted horizontal line is the 0.5*10− 1 threshold and black line is the 0.1*10− 3 threshold. The arrows show the SNPs that are required to explain all the variability present in each trait (JPG 2876 kb) [file 12864_2019_5518_MOESM10_ESM.jpg]

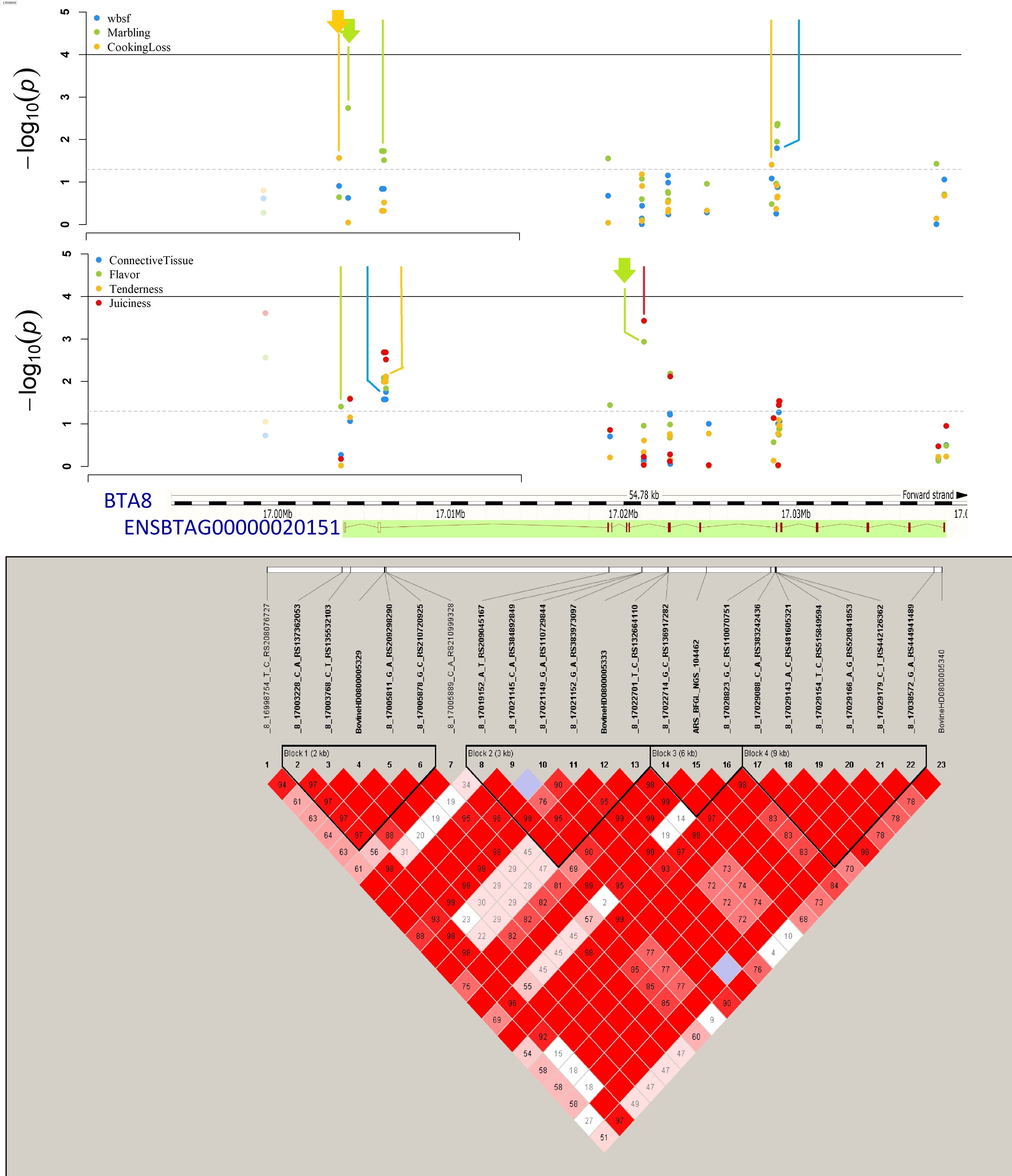

Supplement: Supplementary file 11 — Association analysis for ENSBTAG00000020151 and WBSF, marbling, cooking loss and taste panel in detail. Phenotypes were measured in the longissimus dorsi muscle on Brahman-Angus crossbreed steers. Location of both genes and LD block prediction is presented. Vertical lines highlights the associated uncorrelated SNP by trait. Dotted horizontal line is the 0.5*10− 1 threshold and black line is the 0.1*10− 3 threshold. The arrows show SNPs that could be fitted simultaneously in the association model by each trait. The arrows show the SNPs that are required to explain all the variability present in each trait. (JPG 1750 kb) [file 12864_2019_5518_MOESM11_ESM.jpg]

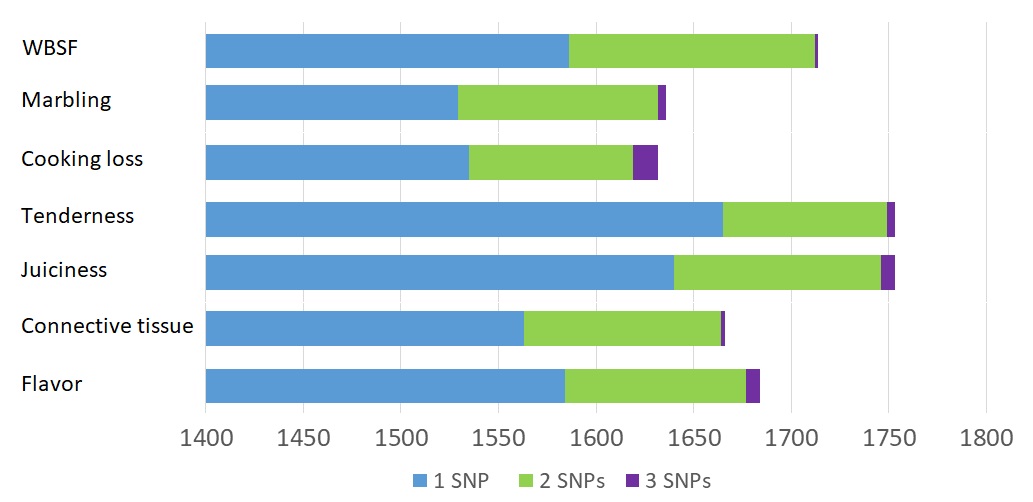

Supplement: Supplementary file 13 — Genes identified by the GWA analysis and included in the final gene lists by trait. These gene lists were used for the gene enrichment analysis and the gene-trait network construction. It is presented the frequency of genes with one or multiple associated uncorrelated SNPs (JPG 69 kb) [file 12864_2019_5518_MOESM13_ESM.jpg]
